# Perceptions of social rigidity predict loneliness across the Japanese population

**DOI:** 10.1038/s41598-022-20561-5

**Published:** 2022-09-27

**Authors:** Ryan P. Badman, Robert Nordström, Michiko Ueda, Rei Akaishi

**Affiliations:** 1 Center for Brain Science, RIKEN, Wako, Saitama 351-0106 Japan; 2 Graduate School of Political Science, Waseda University, Nishi-Waseda, Shinjuku, Tokyo 169-8050 Japan; 3 Faculty of Political Science and Economics, Waseda University, Nishi-Waseda, Shinjuku, Tokyo 169-8050 Japan

**Keywords:** Human behaviour, Risk factors

## Abstract

Loneliness is associated with mental and physical health problems and elevated suicide risk, and is increasingly widespread in modern societies. However, identifying the primary factors underlying loneliness remains a major public health challenge. Historically, loneliness was thought to result from a lack of high-quality social connections, but broader cultural factors (e.g. social norms) are increasingly recognized to also influence loneliness. Here, we used a large-scale survey (*N* = 4977) to assess to what degree the loneliness epidemic in Japan is associated with traditional measures of social isolation (number of close friends), cultural factors (perceptions of social rigidity, as measured by relational mobility), and socioeconomic factors (e.g. income). We confirmed that a lack of close friends is a dominant factor underlying loneliness in Japan. We also found that perceptions of the social rigidity in one’s environment was a major correlate of loneliness. Subjects who perceived lower levels of rigidity in their social environments felt significantly less lonely than those who perceived higher levels of social rigidity, though the association was weak in low income males. Thus, Japanese society and other high social rigidity cultures may need to reflect on the possibility that inflexible traditional norms of socialization are exacerbating loneliness.

## Introduction

“…loneliness is not just about a lack of relationships, but also about the lack of a context or environment in which one can feel at home and oneself”

–Chikako Ozawa-de Silva (p. 216)^1^.

Loneliness is typically defined as a subjective and psychologically painful feeling of lacking meaningful connections with others, and strongly correlates with having insufficient high quality friendships or social contacts^1–3^. A global “epidemic” of loneliness in modern society was declared before the Covid-19 period, with high loneliness scores self-reported by between 10 and 40% of national populations across the United States, Japan, China and Europe^3–7^. Healthwise, loneliness not only causes mental anguish^8,9^, but is also associated with a list of poor physical health outcomes, both long-term and short-term, including dementia, heart disease, higher blood pressure, weakened immune response, and chronic inflammation^2,4,6,10,11^. In fact, the society-level health damage resulting from loneliness (e.g. mortality) has been estimated to be comparable to the damage from obesity and tobacco, with no difference in negative effects seen between objective measures (number of social connections) and subjective measures (self-report emotional scores) of loneliness^10^. Additionally, mounting evidence suggests that loneliness causes structural abnormalities in both white and grey matter areas of the brain, even over short time scales (months to a year)^12,13^, and possibly results in fundamentally different, pathological brain and behavioral states in chronically lonely individuals^2^. Due to worsening social isolation in the Covid-19 pandemic^3^, global attention has increasingly been shifting towards recognition of the often catastrophic society-level effects of untreated loneliness^4^. Generally, though, the factors that are causing and maintaining loneliness in each country may not be the same^7^. For example, compared to European Americans, Asian Americans were found to benefit less from (or even be more stressed out by) receiving explicit social support, possibly due to being raised in harsher East Asian social norms where showing vulnerability is often discouraged^14^. Gender differences in loneliness have been reported to be larger in East Asian cultures as well^15–17^. Unique culture-specific factors have historically been thought to make Japanese nationals particularly susceptible to widespread loneliness^1,18–20^. Indeed, a recent large-scale study showed that 48% of Japanese adults do not talk to anyone about their feelings of loneliness, and 57% of adults feeling loneliness say the condition is due to factors beyond their control^7^. Such stigmatization of loneliness has possibly contributed to the abnormally high suicide rates in Japan^21–23^, where Japan, South Korea and Russia consistently tend to have the highest suicide rates among G20 countries historically^24^. The severity of loneliness within Japan has motivated the recent appointment of a loneliness minister in the national government in order to address the urgent crisis^25^. Current anti-loneliness policy response in Japan however^25^, mirroring international trends^4^, focuses more on number of social connections in clinical counseling, and socialization programs to increase one’s number of friends to ameliorate loneliness (i.e. the size of one’s social network), rather than a focus on understanding how existing social norms may be worsening loneliness (i.e. the structure of one’s existing social networks). Recent work suggests that loneliness, especially in non-WEIRD (Western, Educated, Industrialized, Rich, and Democratic) cultures such as Japan, cannot be understood fully without more research into the latter focus^26,27^.”

Indeed, the main factors underlying widespread loneliness in Japan remain unclear due to loneliness being historically understudied within the country^1^. As one promising direction of research investigation to provide further clarity into correlates of loneliness, cultural factors, such as feeling dissonance with prevalent social norms, have been recently proposed to be major factors underlying loneliness in many countries^1,20,28^. Relative to traditional social isolation and socioeconomic factors, possible cultural factors underlying loneliness have been considerably understudied both globally^20,28–30^ and in Japan^1^. Loneliness resulting specifically from perceptions of relational mobility, a measure of social rigidity (and flexibility)^31^, may particularly be a significant and underappreciated cultural component underlying the loneliness epidemic in Japan, and possibly more broadly in other countries across regions that are low in relational mobility including North Africa, East Asia and the Middle East^20,32–34^. Indeed, Japan is both one of the lowest relational mobility societies in the world^34^ and also is a country with among the highest population-averaged loneliness scores among developed democracies by global loneliness rankings^7^.

**Figure 1:**
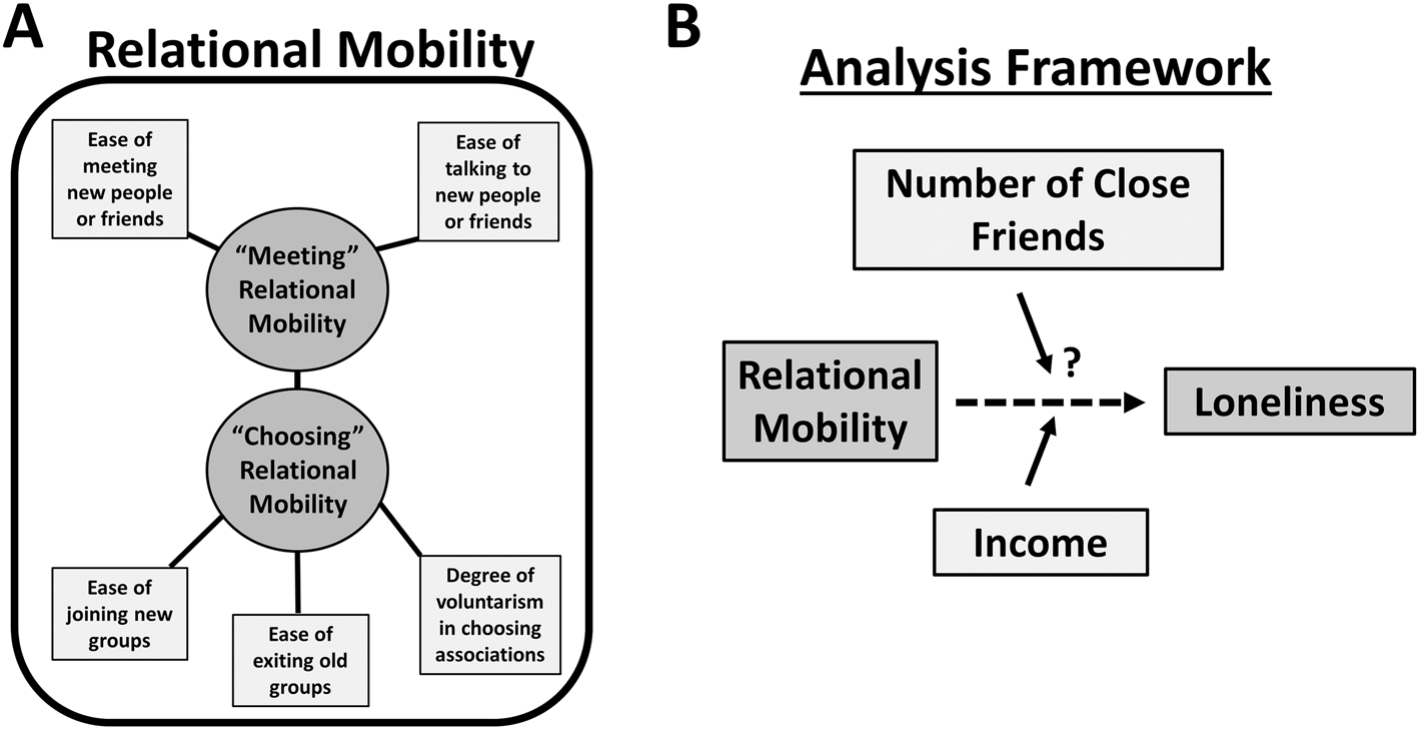
Schematic overview of relational mobility, and the general analysis framework. (**A**) Summary schematic of relational mobility, and the two main “meeting” and “choosing” components that define relational mobility as a construct. We analyze each component of relational mobility separately in all analyses. (**B**) Framework summary of the main variables probed in our analysis.

Relational mobility is a multifaceted socio-psychological variable measuring how easy it is for a members of a society to join and leave social groups voluntarily and establish new social contacts^34,35^ (Fig. 1A), and has been studied at both the individual level and at the aggregate society level^31^. Relational mobility scores at the individual level represent one’s personal, subjective perception of relational mobility in one’s local social environment^36–38^. In contrast, the average relational mobility scores at the aggregate society level are usually interpreted as the de facto relational mobility of a culture, based on the assumption that a culture’s mainstream norms are the “average” of what its constituents’ perceptions of the norms are^34,38,39^. At the individual level, the effects of perceiving lower or higher relational mobility on loneliness remain unclear, though society-level results have linked higher average loneliness with lower relational mobility across cultures^26^. One individual-level explanation is that perceptions of low relational mobility may lead people to believe that most of the others in their environment prefer more rigid and formal social interactions even if this is not actually the case^39^, hindering development of more intimate and meaningful social connections^1,20,26,38^. Alternatively (or in parallel), having more personal choice in forming relationships (higher relational mobility) may make people value and invest more in their chosen social connections, compared to the situation where social connections are just passively assigned in rigid social environments (lower relational mobility)^32,33,36,40,41^. However, relational mobility has been historically predominantly reported as a static value per culture or nation, leading relational mobility researchers to write calls encouraging a better understanding of how within-country differences in perceptions of relational mobility relate to individual-level outcomes in a given country, yet little progress has been made in this direction^31,42^. Therefore, more in-depth investigation into the effects of perceptions of relational mobility on loneliness at the individual level is necessary to understand the importance of this psychological construct, and we have sought to provide insight towards this direction in this work. We hope that more broadly such insight encourages consideration of the individual-level effects of perceptions of relational mobility (or perceptions of other traditional macroscale cultural variables^38^) within future cultural psychology research, and international policy and clinical research.

To determine to what extent traditional social isolation measures (number of close friends) versus cultural factors and social norms (relational mobility) are contributing to loneliness at the individual-level, during the Covid-19 pandemic period, we have conducted a large-scale general population survey study across all prefectures in Japan, collected in three data collection waves from December 2021 to February 2022 (*N* = 4977) (“Methods”). Our survey measured self-reported loneliness, number of close social connections, perceptions of relational mobility, socioeconomic status (income) and then other standard demographics as control variables (Methods, Supplementary Information). Our research team included native experts in Japanese culture to aid in the culture-specific complexities and nuances that were expected to present during the interpretation of the results. We predicted that there would be two major correlates of loneliness in the Japanese general population: (1) a lack of close, high-quality friends or intimates (i.e. social isolation) and (2) the perceptions of the general difficulty in joining and leaving social groups (i.e. relational mobility). To best utilize our large dataset, we probed this hypothesis primarily by using linear regression analyses, which we complemented with visualization-focused secondary analyses, including deeper investigation into high loneliness sub-populations with further statistical analyses and conditional inference trees. Last, to help investigate conflicting results for gender differences in loneliness internationally^15^, and in Japan^17,18,43^, we conducted additional analyses that separated males and females to help reveal possible gender differences in social environments. The overall applied goal of our large general population study was to identify strong correlates of loneliness within Japan to help inform important future targets for anti-loneliness policy intervention, while the overall scientific goal was to understand the within-country relationship between perceptions of relational mobility and subjective loneliness in Japan (Fig. 1).

## Results

The primary goal of our main analyses was to find to what degree loneliness in Japan correlates with traditional measures of social isolation versus the cultural factor of social rigidity (relational mobility), and a secondary goal was to test the mediating effects of gender and age within the primary analysis. Linear regression was the central method used to conduct this analysis. To further expand upon the regression results, we also probed more visually in statistical sub-population analysis to see how different levels of perceptions of relational mobility modulate loneliness on top of the more traditionally considered base variables of (1) numbers of close friends and (2) income levels (Fig. 1B). Within all analyses, to aid interpretation following prior work^34^, we divided relational mobility into its two main sub-components: “choosing” and “meeting” types of relational mobility (Fig. 1A, “Methods”).

### Regression analysis of the factors underlying loneliness

In social psychology literature, the largest universal component underlying loneliness has consistently been a lack of high-quality social connections^1,2^. Then, in social sciences and economics literature, researchers found that socioeconomic status, especially household income, was one of the largest universal economic-related confounding variables underlying loneliness^44^. This income trend has also been reported in Japan^18^. Thus, to quantitatively investigate the association between relational mobility and loneliness relative to the primary explanatory variable of number of friends (social isolation), a primary confounding factor of income (socioeconomic status), and a list of socioeconomic and demographic variables as possible secondary confounding variables motivated from prior work^18^, we performed linear regression (“Methods”). The descriptive summary of the main analysis variables is provided in Table 1, while the extended descriptive summary for the full survey is provided in Supplementary Table S1, and the distributions of the relational mobility scores (meeting and choosing) are provided in Supplementary Fig. S1.

**Table 1:** Descriptive statistics from the survey data for the primary analysis variables.

		Males			Females		
		*N*	%*	*Loneliness* (mean, 95% CI) (0–30 scale)	*N*	%*	*Loneliness* (mean, 95% CI) (0–30 scale)
Age groups	20–29	590	23.8	14.4 (14.1–14.8)	586	23.5	13.2 (12.8–13.6)
	30–39	375	15.1	14.5 (14.0–14.9)	373	14.9	13.3 (12.8–13.8)
	40–49	400	16.1	14.4 (13.9–14.9)	392	15.7	13.9 (13.4–14.4)
	50–59	372	15.0	14.8 (14.3–15.2)	373	14.9	13.1 (12.6–13.6)
	60–69	393	15.9	13.0 (12.5–13.4)	401	16.1	12.2 (11.7–12.7)
	70+	350	14.1	12.2 (11.7–12.7)	372	14.9	10.7 (10.2–11.2)
Income (million yen)	< 2	264	10.7	15.7 (15.1–16.3)	365	14.6	13.8 (13.3–14.4)
	2–4	617	24.9	14.3 (13.9–14.6)	700	28.0	12.8 (12.4–13.2)
	4–6	638	25.7	14.0 (13.6–14.3)	609	24.4	12.7 (12.3–13.1)
	6–8	440	17.7	13.6 (13.2–14.1)	405	16.2	12.3 (11.8–12.7)
	8–10	251	10.1	12.6 (12.1–13.1)	203	8.1	12.2 (11.5–12.9)
	10+	270	10.9	13.1 (12.6–13.6)	215	8.6	12.6 (12.0–13.2)
Number of close friends	0 friend	577	23.3	17.0 (16.6–17.3)	385	15.4	16.8 (16.3–17.3)
	1 friend	329	13.3	15.2 (14.9–15.5)	351	14.1	14.9 (14.4–15.3)
	2 friends	528	21.3	13.9 (13.5–14.2)	605	24.2	13.0 (12.7–13.4)
	3 friends	395	15.9	12.6 (12.2–13.0)	459	18.4	11.5 (11.1–11.9)
	4+ friends	651	26.3	11.5 (11.1–11.8)	697	27.9	10.1 (9.8–10.4)

*The “%” column is calculated as the percent out of the total number of subjects within the gender group (*N* = 2480 for males, *N* = 2497 for females). Combined-gender, full population results are in Supplementary Table S1. 95% confidence intervals (CIs) around the mean are given in parentheses in the loneliness columns.

We found that a higher number of close friends and perceptions of higher relational mobility (both meeting and choosing relational mobility) were among the strongest correlates of lower loneliness (Tables 2 and 3), even when controlling for a long list of diverse socioeconomic variables including income, employment status, age, residential mobility, etc. The standardized regression coefficients of both increasing number of close friends and perceptions of higher relational mobility were each approximately the same magnitude as the coefficients from being married in reducing loneliness (marriage was included as an important social-related reference variable known to correlate with lower loneliness^43,45^). Furthermore, there were significant interactions between number of close friends with gender and with age in predicting loneliness, but only interactions with relational mobility (choosing) and gender, in predicting loneliness (Table 3). See Supplementary Tables S2 and S3 for the extended regression results of all secondary confounding variables (abbreviated Tables 2 and 3 are shortened from the extended versions of Supplementary Tables S2 and S3).

**Table 2:** Abbreviated OLS regression of factors underlying loneliness in Japanese general population sample.

Variables	Loneliness	
	(coefficient)	(95% CI)
Number of close friends	− 0.385***	(− 0.409 to − 0.360)
Relational mobility (meeting)	− 0.139***	(− 0.164 to − 0.114)
Relational mobility (choosing)	− 0.126***	(− 0.152 to − 0.099)
Income	− 0.036**	(− 0.063 to − 0.008)
Gender (Female: 1, Male: 0)	− 0.197***	(− 0.249 to − 0.145)
Age	− 0.137***	(− 0.174 to − 0.099)
Married	− 0.227***	(− 0.292 to − 0.162)
Observations	4977	
R-squared	0.310	
Demographic controls	See Table S2	
Regional dummies	See Table S2	

*p < 0.10; **p < 0.05; ***p < 0.01. Regression coefficients for factors underlying loneliness, with standardized regressors. Negative coefficients correspond to lower loneliness. 95% confidence intervals (95% CIs) are given in parentheses. Only coefficients for the most relevant independent variables are shown, for the full regression table see Supplementary Table S2.

**Table 3:** Abbreviated OLS regression of factors underlying loneliness in Japanese general population sample, with moderator variables of gender and age.

Variables	Loneliness	
	(coefficient)	(95% CI)
Number of close friends	− 0.349***	(− 0.382 to − 0.316)
Relational mobility (meeting)	− 0.128***	(− 0.166 to − 0.091)
Relational mobility (choosing)	− 0.084***	(− 0.123 to − 0.045)
Income	− 0.037***	(− 0.064 to − 0.010)
Gender (Female: 1, Male: 0)	− 0.197***	(− 0.248 to − 0.145)
Age	− 0.134***	(− 0.171 to − 0.096)
*Interaction Term*: Number of Close Friends & Gender	− 0.077***	(− 0.125 to − 0.029)
*Interaction Term*: Number of Close Friends & Age	− 0.046***	(− 0.070 to − 0.022)
*Interaction Term*: Relational Mobility (Meeting) & Gender	− 0.014	(− 0.063 to 0.035)
*Interaction Term*: Relational Mobility (Meeting) & Age	− 0.015	(− 0.040 to 0.009)
*Interaction Term*: Relational Mobility (Choosing) & Gender	− 0.072***	(− 0.121 to − 0.023)
*Interaction Term*: Relational Mobility (Choosing) & Age	− 0.016	(− 0.040 to 0.008)
Married	− 0.233***	(− 0.298 to − 0.169)
Observations	4977	
R-squared	0.316	
Demographic controls	(see Table S3)	
Regional dummies	(see Table S3)	

*p < 0.10; **p < 0.05; ***p < 0.01. Regression coefficients for factors underlying loneliness, with standardized regressors. Negative coefficients correspond to lower loneliness. 95% confidence intervals (95% CIs) are given in parentheses. Only coefficients for the most relevant independent variables are shown, for the full regression table see Supplementary Table S3.

Additionally, certain exploratory age- and gender-dependent findings picked up in further age- and gender-grouped supplementary regression analyses (Supplementary Tables S4 and S5) may warrant follow-up investigation in future work, at least in the context of Japan (e.g. full-time employment significantly correlated with lower loneliness for middle-aged females, residential mobility correlated with higher loneliness and religiousness correlated with lower loneliness for older subjects only, amount of weekly free time affects younger versus older male loneliness differently, single parent males have higher loneliness, etc.). Such trends are beyond the scope of our current work to explore however.

**Figure 2:**
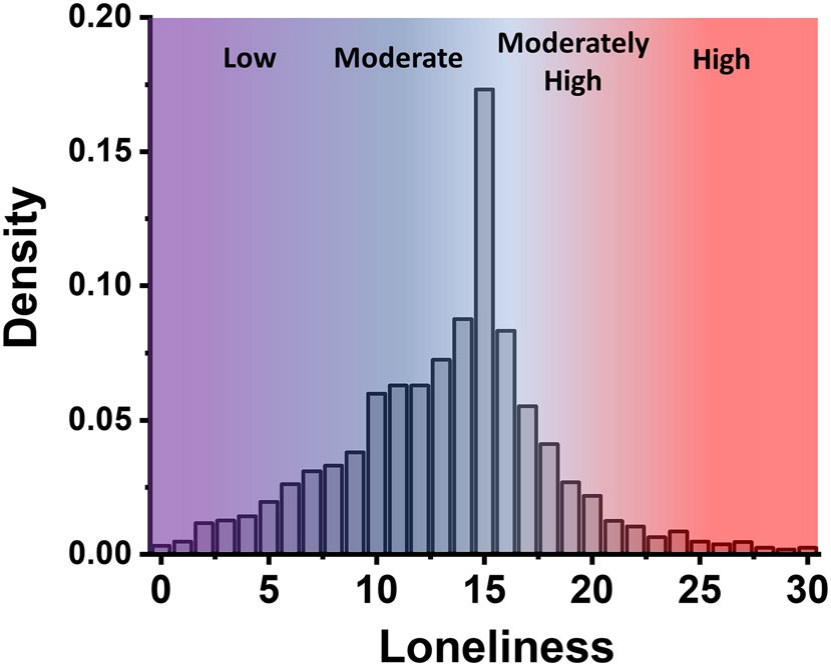
Summary of loneliness scores across a general population survey sample in Japan. Fraction of the respondents with each possible loneliness score. Loneliness was calculated based on the SF-10 loneliness scale, with ten 4-point questions and a total range of 0–30 points, and higher scores representing higher loneliness. Overlaid are approximate positions of four typical loneliness scoring categories of “Low”, “Moderate”, “Moderately High”, and “High” that are often used in the SF-20 loneliness scale (not used in this work), which has a score range of 0–60. In the SF-20 scale, 0–14 denotes a low degree of loneliness, 15–29 a moderate degree of loneliness, 30–44 a moderately high degree of loneliness, and 45–60 a high degree of loneliness. Thus, approximate scoring boundaries within the SF-10 scale that was used in this study would be 0–7 (low), 7.5–14.5 (moderate), 15–22 (moderately high), and 22.5–30 (high). No official categorization exists yet in the SF-10 scale.

Furthermore, to aid in visualization and interpretation of the dependent variable, the distribution of scores for loneliness across our Japanese general population sample is presented in Fig. 2. Self-reported mental health reports for loneliness were alarming across our Japanese general population sample, with a large amount of the population in higher loneliness categories. Approximate categorical labels were overlaid over our SF-10 loneliness results (scored 0–30) based on analogous positions of the most common categorization used in the older SF-20 loneliness scale (scored 0–60, not used in this study)^46^. In typical interpretations for the SF-20 scale, 0–14 denotes a low degree of loneliness, 15–29 a moderate degree of loneliness, 30–44 a moderately high degree of loneliness, and 45–60 a high degree of loneliness^46^. Thus, if the SF-20 category ranges are halved to match the SF-10 ranges, approximate boundaries in the SF-10 scale would be 0–7 (low), 7.5–14.5 (moderate), 15–22 (moderately high), and 22.5–30 (high), however no official categorization yet exists for the SF-10 scale^47^. Note that the original continuous SF-10 loneliness scores, not categorically binned values, were used in all quantitative analyses in this work however.

### Visual and statistical investigation into the effects of relational mobility, compared to number of friends, on loneliness

**Figure 3:**
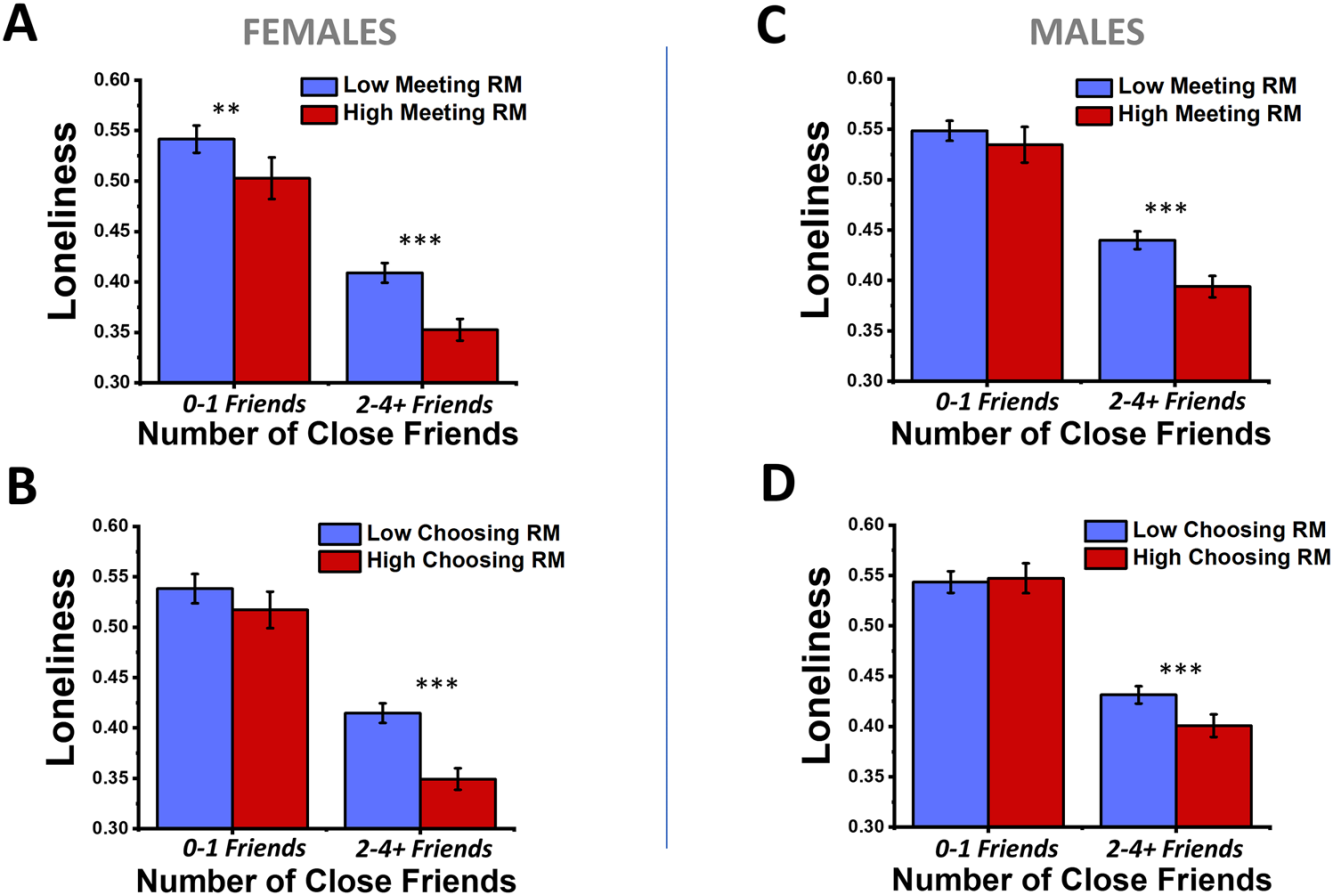
Plots of how perceptions of relational mobility and number of close friends associate in predicting loneliness for males and females. For females (**A**, **B**) versus males (**C**, **D**), and the meeting type of relational mobility (**A**, **C**) and choosing type of relational mobility (**B**, **D**), bar plots are presented showing the mean loneliness for major sub-populations of subjects that have either 0–1 close friends or 2–4 + close friends, then further divided into subjects that have either lower (blue) or higher (red) perceptions of relational mobility than the average relational mobility value (continuous scores) reported across the entire survey sample. Within each bar plot, for each number-of-close-friends options, two-way ANOVA followed by Bonferroni-corrected pairwise tests were used to identify whether higher and lower relational mobility sub-groupings had significantly different means within each sub-population. For the pairwise tests within each number-of-close-friends category, labels were: * p < 0.05, ** p < 0.01, ***, p < 0.001. Loneliness scores are the continuous raw scores normalized by the max value of 30. Error bars are 95% confidence intervals.

Next, we wished to probe how varying levels of perceptions of relational mobility in one’s social environment modulate loneliness on top of the effects of a given number of close friends on loneliness (Fig. 3). Since there were interaction effects between relational mobility and gender, but not relational mobility and age (Table 3), and because gender differences in loneliness have been reported to be important in East Asian cultures as well^15–17^, we have further separated out the sample into male and female in the visual-focused analyses.

For the first secondary analysis, we visualized how each type of relational mobility interacts with the number of close friends, for males and females separately, using sub-population analysis (Fig. 3). Note in the summary of descriptive statistics, 23.3% of men and 15.4% of women reported having no close friends at all, while 13.3% of men and 14.1% of women reported having only one close friend (Table 1). Thus, approximately one third of the adult Japanese population is in a socially isolated status. As previously reported^2,7^, the number of close friends had a large association with loneliness scores (Table 1, Fig. 3). In addition to this correlation between the quantity of close social connections and loneliness, we also found significant additional correlations between loneliness and perceptions of relational mobility. Especially for subjects who have two or more close friends, a statistically significant drop in loneliness was seen in subjects who perceive higher relational mobility for both choosing and meeting types of relational mobility. This finding was observed for both genders (Fig. 3). The largest difference of sub-population means in loneliness between higher and lower relational mobility groups, was a difference of 6.5% of the full range of the SF-10 loneliness scale, for females with 2–4+ friends (Fig. 3B). In contrast, the differences in population means resulting from lower versus higher number of close friends was ~ 10% of the loneliness scale range across genders.

More detailed statistical tests were then performed to explore the interaction effects of relational mobility and number of close friends on loneliness for females and males, using two-way ANOVAs. The two-way interactions between number of close friends and relational mobility were not significant (*p* = 0.219) for meeting relational mobility scores of females, significant (*p* = 0.001) for choosing type relational mobility scores of females, significant (*p* = 0.009) for meeting relational mobility scores of males, and significant (*p* = 0.004) for choosing type relational mobility scores of males. See Supplementary Tables S6–S9 for the full ANOVA results for this analysis. Thus, in brief, the number of close friends and perceptions of relational mobility interact in predicting loneliness in the choosing type for both males and females, and in the meeting type just for males. Therefore, both traditional social isolation factors and cultural factors underlying loneliness must be simultaneously considered to understand loneliness in Japanese society.

### Visual and statistical investigation into the effects of relational mobility, compared to income, on loneliness

**Figure 4:**
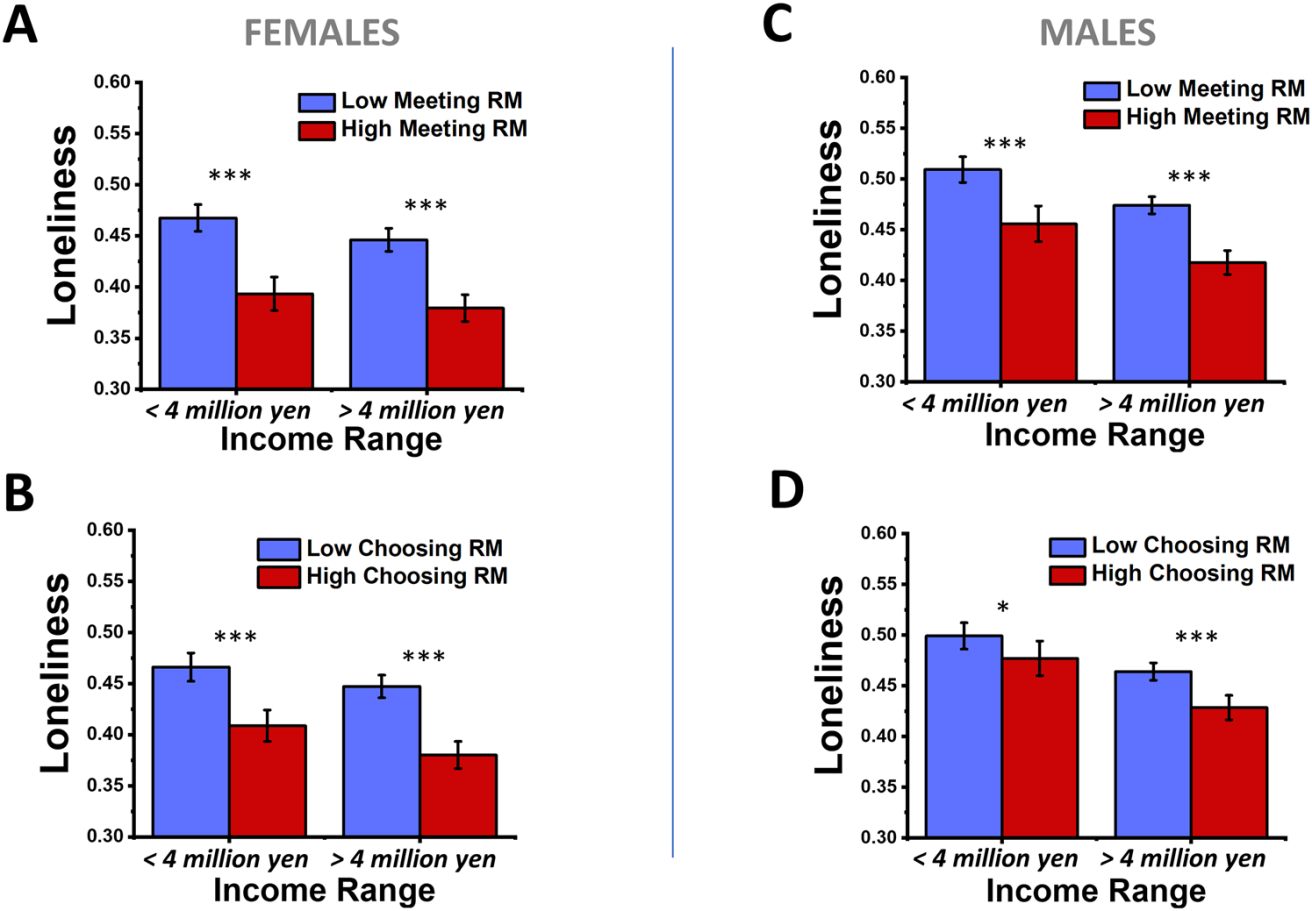
Plots of how perceptions of relational mobility and income associate in predicting loneliness for males and females. For females (**A**, **B**) versus males (**C**, **D**), and the meeting type of relational mobility (**A**, **C**) and choosing type of relational mobility (**B**, **D**), bar plots are presented showing the mean loneliness for major sub-populations of subjects that have either less than or higher than four million yen of annual household income, then further divided into subjects that have either lower (blue) or higher (red) perceptions of relational mobility than the average relational mobility value reported across the entire survey sample. Within each bar plot, for each of the income options, two-way ANOVA followed by Bonferroni-corrected pairwise tests were used to identify whether higher and lower relational mobility sub-groupings had significantly different means within each sub-population. For the pairwise tests within each income, labels were: * p < 0.05, ** p < 0.01, ***, p < 0.001. Loneliness scores are the continuous raw scores normalized by the max value of 30. Error bars are 95% confidence intervals.

We next probed how varying perceptions of relational mobility levels in one’s social environment modulate loneliness for subjects of different income levels. As in the previous section, we visualize how each type of relational mobility interacts with income, for males and females separately, using sub-population analysis (Fig. 4).

Within Fig. 4, having both low income and perceiving lower relational mobility of both types correlates with a higher degree of loneliness, but within a given income range the perceptions of relational mobility also correlates with relative loneliness levels. However, there was a gender-specific finding that the potentially protective effects of higher relational mobility perceptions were potentially weaker for low income males. Additionally, low income males have the highest baseline loneliness (Fig. 4C, D), confirming a prior Japanese study^18^. However, for both genders, having higher income while also perceiving higher relational mobility in one’s social environment correlates with the lowest amount of loneliness.

Two-way ANOVA statistical tests were then performed to explore the effects of relational mobility and income on loneliness for females and males. Unlike the number of close friends and perceptions of relational mobility, income and perceptions of relational mobility were found to have statistically independent but individually significant effects on loneliness (significant main effects of each factor: *p* values ranged from < 0.001 to 0.042). However, two-way interaction effects between income and relational mobility were not significant in any gender or relational mobility type (*p* values ranged from 0.296 to 0.813 for the two-way interactions of relational mobility and income). See Supplementary Tables S10–S13 for the full ANOVA results for this analysis.

### Visual and statistical investigation into the combined effects of relational mobility, number of friends, and income on loneliness

**Figure 5:**
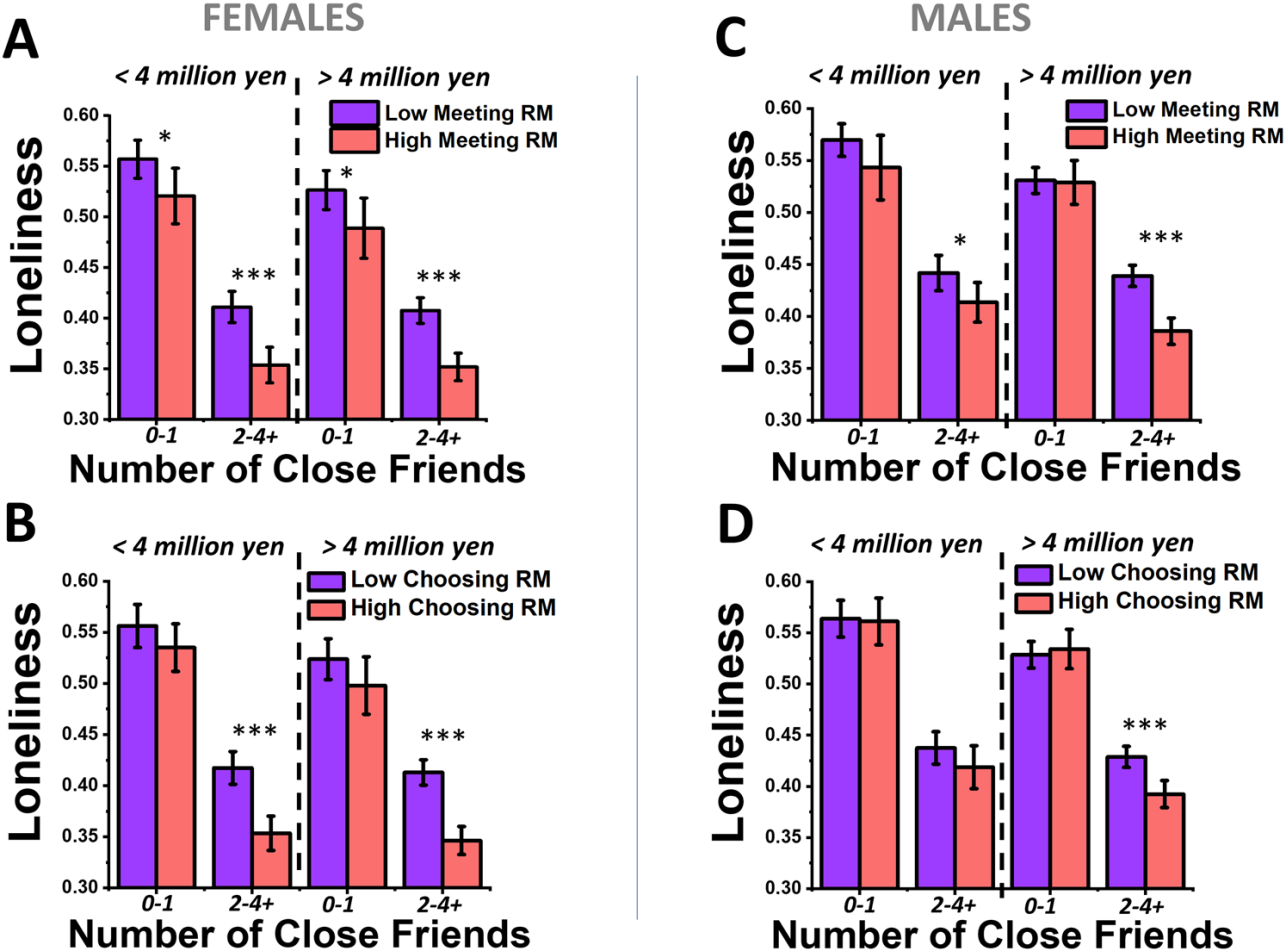
Summary of how perceptions of relational mobility correlate with loneliness when controlling for both number of close friends and income. Bar plots are presented showing the mean loneliness for major sub-populations that have either less than or higher than four million yen of annual household income, *and* also either 0–1 close friends or 2–4+ close friends, and then further divided into subjects that have either lower (purple) or higher (salmon pink) perceptions of relational mobility than the average relational mobility value reported across the entire survey sample. Within each bar plot, for each of the low/high income ranges plus low/high number-of-close-friends categories, two-way ANOVA followed by Bonferroni-corrected pairwise tests were used to identify whether higher and lower relational mobility sub-groupings had significantly different means within each sub-population. For the pairwise tests within each number of close friends and income sub-population, labels were: * p < 0.05, ** p < 0.01, ***, p < 0.001. Loneliness scores are the continuous raw scores normalized by the max value of 30. Error bars are 95% confidence intervals.

We have separately examined the effects of number of close friends and income with the effects of relational mobility on loneliness. In the prior analyses, we found the effects of relational mobility on loneliness existed as an interaction effect with the number of close friends, and as an independent effect from income. Still, there is a possibility that combined interaction effects of number of close friends and income can explain away at least part of the effects of the relational mobility on loneliness^48^. As a last secondary analysis, therefore, we asked whether it is possible that the number of close friends and income may interact with each other in a way that confounds our interpretation of the effects of relational mobility on loneliness. Thus, following and combining the analysis logic of Figs. 3 and 4, we further divided subjects into sub-populations of those who had lower numbers of close friends *and* lower income, those who had higher numbers of close friends *and* lower income, those who had lower numbers of close friends *and* higher income, and those who had higher numbers of close friends *and* higher income, to examine differential patterns in loneliness across these sub-populations, in both genders (Fig. 5). For each of these sub-populations, we analyzed the effects of relational mobility on loneliness.

The effects of relational mobility perceptions on loneliness were strongest and most consistent in both genders for subjects with sufficient numbers of friends (2–4+) (Fig. 5). For males, these patterns are observed in the sub-population of middle-to-high income but not in the low income group. Thus, low income males appeared to be a possible outlier group with the regard to the link between relational mobility perceptions and loneliness. For further analysis in this direction, three-way ANOVA statistical tests were performed to explore the three-way interaction effects of perceptions of relational mobility, number of close friends and income on loneliness for females and males. In brief summary, for males there was a trend of three-way interaction effect between income and number of close friends and relational mobility (*p* = 0.051) for meeting relational mobility, but no significant trend (*p* = 0.290) for choosing relational mobility whereas for females there were no significant three-way interactions (*p* = 0.929 and *p* = 0.944 for the three-way interactions involved with meeting and choosing relational mobility respectively). See Supplementary Tables S14–S17 for the full ANOVA results for this analysis.

This combined analysis in Fig. 5 solidifies our interpretations of the major secondary results. We found that (1) the association between perceptions of relational mobility and loneliness was strongest for subjects who have 2–4+ close friends across the sub-populations, while (2) the association between relational mobility perceptions and loneliness was weakest for subjects who have 0–1 close friends across the sub-populations. The latter finding (2) makes sense, given that lacking sufficient numbers of close friends has long been established to be the primary driver of loneliness in social psychology literature^1,2^. The former result (1) may help explain recently reported discrepancies to the traditional loneliness picture, such as the findings that loneliness often continues to be reported in individuals even when they report having enough social connections^20,28^. As an additional interpretation, perceptions of rigidity in social interactions may be less salient to those who lack social connections, given that relational mobility is a fundamentally social construct, which can exist only where there are social connections^31^. Thus, we provide evidence supporting the idea that cultural factors (social norms) may partially drive loneliness in Japanese society.

### Conditional inference tree analysis of the factors underlying loneliness

Last, we performed a supplementary analysis to identify clustered characteristics of sub-populations which have an increased risk of high loneliness. Conditional inference trees use statistical tests to find which sets of explanatory variables and specific values of those variables, rather than effects of single variables, delineate sub-populations^49,50^. This approach allows for investigation of which sets and values of explanatory variables cluster together in defining sub-populations of various levels of loneliness for example. Conditional inference trees rank which explanatory variables best predict dependent variable response, with more important variables towards the top of the tree and clustered variables within the same branch of a tree. Thus, we use this data-driven conditional inference tree approach to identify how differing number of friends, differences in perceptions of each type of relational mobility, and various parameter values of socioeconomic variables cluster to define loneliness levels across the general Japanese population sample.

Following the gender effects observed in the regression analyses (Table 3, Supplementary Tables S4 and S5) and in the sub-population analysis (Figs. 3, 4 and 5), we analyze males and females separately. In the results presented by forest importance factor plots, to validate the conditional inference tree technique, we confirmed that the number of close friends was the dominant factor for predicting loneliness (Supplementary Fig. S2). Further agreeing with our primary results, we also found that perceptions of relational mobility had a strong association with loneliness. Relational mobility had approximately equal importance to the factor of being married and it had a greater importance than almost all other socioeconomic variables, within random forest results of 1000 conditional inference trees (Supplementary Fig. S2). As a new result revealed by the conditional inference tree approach however, per gender certain parameter values of the number of close friends, relational mobility, and age were found to cluster together within branches of the inference trees to predict loneliness (Supplementary Figs. S3 and S4), showing associative trends beyond those that regression (which treats single variables as independent) can reveal, and that may warrant investigation in future work. Thus, anti-loneliness policy interventions may need to be tailored towards specific sub-populations in Japan in targeted ways.

## Discussion

Confirming prior work^7,18^, we presented evidence for widespread moderate-to-high loneliness across Japan. This high level of loneliness should be considered alarming by both local communities and the national government in Japan. The most dominant factor for predicting lower loneliness in our data was the number of close friends, agreeing with foundational loneliness theory^1,2^. Having zero or only one close friend predicted more extreme loneliness across both genders and income levels. The number of close friends was verified as the most important variable for predicting loneliness in both linear regression and conditional inference tree analysis as well. As our core novel result beyond the number of close friends though, we also found that perceptions of the relational mobility in one’s environment was a potentially major influence on how much loneliness people feel for a given social context, especially once subjects had an adequate number of close friends. Subjects who perceived higher levels of relational mobility in their social environment felt significantly less lonely than those who perceived lower levels of relational mobility as a general correlation. Additionally, there was high consistency in results between meeting and choosing types of relational mobility^34^, supporting the validity of the relational mobility construct in general^31^.

Extending from our overall results and following prior work^20,28^, loneliness likely then has at least two major separate components within Japan, the traditionally considered social isolation component reflecting the number of quality social relationships one has (number of close friends), and a second cultural (social norms) component. Here, the measured cultural factor of relational mobility reflects current and future considerations of how easy it is to form desired social relationships in one’s social environment (Fig. 1A). One qualitative individual-level explanation of our findings is that people who have perceptions of high social rigidity (low relational mobility) may believe that most people in their environment prefer more rigid and formal social interactions even if this is not actually the case^39^. This mistaken belief may discourage people from forming more intimate relationships^1,20,26,38^. Additionally, as prior work has shown that having more voluntary choice in forming relationships may make people value and invest more in their chosen social connections, relational mobility as a construct may also partially capture the degree of voluntarism that people believe they have in their social environment^32,33,36,40^. Furthermore, another possible interpretation of these perceived relational mobility scores at the individual-level is that the scores partially reflect subjects’ *hopelessness* (low perceived relational mobility) or *hopefulness* (high perceived relational mobility) about their future social prospects^51^. If one has little-to-no close friends, but feels they can easily meet new people and/or flexibly join new social groups, then this reduces feelings of loneliness relative to the situation of having little-to-no close friends while feeling there is no hope for meeting new friends or joining new social groups. Last, lower relational mobility in a social environment (at least at the community scale and national scale) has also been found to cluster with both worse perceived inequality (i.e. more unfairness) and lower general trust^31,52,53^. This association is conceptually explained by the idea that communities form more rigid social hierarchies when people trust each other less and/or perceive more unfairness or risk in the environment^34,40^. Lower general trust may also degrade the quality or intimacy of social interactions if it overlaps with low relational mobility. Prior work has argued that these situations exist in Japan^31,40^. Future work should consider investigating the interaction between general trust and relational mobility more closely.

Next, lower income males (even with higher numbers of close friends) are a possible outlier sub-population in our results (Fig. 5). The data in this sub-population potentially goes against the core relational mobility patterns we observed, with loneliness correlated with relational mobility perceptions to a statistically weaker degree for these lower income males than for other sub-populations. This tentative finding is beyond the scope of this work to explain in detail, but may be due either to the Japan-specific *hikikomori* (abnormal and prolonged social withdrawal) known to be most prevalent within the low income male population^54^, due to traditional Japanese culture expectations that put more pressure on men to be the breadwinners of their household thus making them more income-focused rather than relation-focused^19,55^, or due to the sharply stratified seniority systems that are known to affect males the most in Japanese culture, which may be true both in social life and in the workplace^19^.

For planning policy interventions, following prior qualitative research^1,21^, we posit that a lack of high quality social relationships, and additionally dissatisfaction about or dissonance with current norms of social rigidity, are complementary problems that must both be faced to combat loneliness in Japanese society.

Japanese-specific gender discrimination and segregation may further compound social rigidity problems for both genders in distinct ways^56,57^, consistent with gender-specific differences we observed especially for the relationship between economic-related variables and loneliness. Moving forward, to help design interventions in this area, we suggest that more in-depth qualitative research (e.g. subject interviews) be performed to better understand how subjects interpret relational mobility as manifesting in their daily lives, as to date relational mobility is primarily studied by quantitative researchers in brief survey response format^34^.

Last, relational mobility has been historically reported as a fixed average value per culture or nation that does not readily change over time, and may be a result of an equilibrium state of a balance of advantages and disadvantages at the society-level^34,35^. However, relational mobility researchers have written calls encouraging a better understanding of how within-country differences in perceptions of relational mobility relate to individual-level outcomes in a given country, yet little progress has been made in this direction^31,42^. Here, in an effort towards this proposed direction, we have shown that within Japan the individual-level variability in relational mobility perceptions significantly correlates with loneliness, even when controlling for number of social connections, socioeconomic factors and gender. Such a result would not emerge if we had treated relational mobility as an averaged macroscale variable as is usually done, and thus highlights the general need for developing multiscale interpretations and theories of intersubjective social phenomena like relational mobility and loneliness^38,58,59^. Therefore, our results may convey an important general message for the field of cultural psychology and international policy and clinical research, especially for researchers seeking to better understand non-WEIRD cultures^27^. Since Hofstede’s seminal research on his initial set of national-level, macroscale cultural dimensions in the 1980s^60^, much of the field’s historical approach has at least partially assumed individuals from a given culture share approximately the same macroscale cultural variable value on average, and interpret behavior or outcomes based on this “passport” assumption^61,62^. Recent critiques have pointed out that such an approach may risk neglecting the large variability and dynamics at the individual or regional-level in cultural norms and beliefs (especially as globalization has progressed)^58^, may risk ignoring the influence of institutional behavior and state-encouraged social norms that are separate from cultural norms (e.g. South versus North Korea as an obvious example)^63^, and may underestimate or ignore multi-level interactions between the individual and society-level^58,59,64,65^. Thus, we hope our work encourages more consideration of the individual-level effects of perceptions of relational mobility (or perceptions of other traditional macroscale cultural variables^38^) within future cultural psychology research, and international policy and clinical research.

Overall, the potential benefits we find from pockets of higher relational mobility within the overall low relational mobility Japanese society^34^, suggests that Japanese social groups and individuals broadly may need to reflect about becoming more socially flexible and inclusive to combat loneliness and poor mental health in their communities, even at the cost of going against some traditional social norms. Such reflection is important, as untreated loneliness is now established to result in massive psychological and physical health costs in modern societies^2,4^ and prior work suggests loneliness continues to worsen throughout Japan^7,18^. As we have found a consistent correlational link between relational mobility perceptions and loneliness across gender and other demographics in Japan in this work, interventions that address potentially problematic social norms in society may be important to consider in future anti-loneliness policy.

## Conclusion

Loneliness was reported to be a significant problem even before Covid-19 in modern Japanese society^7^ and worldwide^2,4^. Here we confirm severity of loneliness during the late Covid-19 pandemic (December 2021–February 2022). We find the main social correlates of loneliness in the general population of Japan to be twofold: a lack of sufficient numbers of close friends as the strongest correlate of higher loneliness, and then perceptions of rigidity in the social environment as a significant secondary correlate. These findings held even after controlling for socioeconomic conditions such as income. Thus, in addition to the current anti-loneliness policy approach of improving welfare systems and increasing clinical referrals for counseling^25^, Japanese society and other low relational mobility cultures may need to address broader sociocultural problems stemming from rigid traditional norms of socialization. We hope our results can help encourage action and policy changes at both the community and national level, to help mitigate the damaging social, medical and economic effects of untreated loneliness.

## Methods

### Data collection

We conducted an online cross-sectional survey of the Japanese general population in three waves in 2021 and 2022 (December 2021, January and February 2022) using a commercial survey company, the Survey Research Center (https://www.surece.co.jp/). The Survey Research Center uses internal prescreening protocols to maintain a high-quality subject pool for survey-based research in Japan, and has supplied subjects for Japanese loneliness research in prior work^18,66^. The three collection waves are comprised of the following data collection periods: December 21st–24th 2021 (*N* = 1848), January 14th–24th 2022 (*N* = 1540), and February 1st–7th 2022 (*N* = 1600). The survey responses were collected by the Survey Research Center over the internet. The sample consisted of the adult population (age 20+) and those living in Japan (*N* = 4988).

Sampling criteria in our study included 10-year age groups that were each well-represented in our final sample (Table 1, Supplementary Table S1). Equal representation of both genders was a further sampling requirement, with 49.7% of respondents being male and 50.0% being female. The rest (*N* = 11 “other”) were unfortunately excluded from the analysis due to inadequate statistics and the importance of gender in our study, thus reducing the original *N* = 4988 to the *N* = 4977 used here. Then mostly equally weighted sampling by Japanese geographical region was the last constraint on data collection. The respondents could participate only in one of the three survey waves. The participants were informed of the purpose of the study, were allowed to exit the survey at any point, and were monetarily compensated for their participation. The data were collected and recorded completely anonymously.

### Main analysis variables

#### Loneliness

Loneliness is a subjective self-report of the degree of feeling socially isolated and disconnected, and we use the Japanese version of the ten-item UCLA loneliness scale (SF-10) to measure loneliness^47,67^. The scale consists of ten items and the choices for each item are: *never* (0), *rarely* (1), *sometimes* (2), *always* (3). These scores were aggregated across the ten items to calculate the total score (range 0–30), and a higher score indicates higher levels of loneliness. The scale has a high internal consistency (Cronbach’s alpha = 0.82). During analysis, loneliness was kept as the continuous summed score from all ten questions.

#### Relational mobility

We administered the standard 12-item scale of relational mobility^31^. The “choosing” type of relational mobility with the standard seven “choosing” questions from the original relational mobility scale, and the “meeting” type of relational mobility with the standard five “meeting” questions from the original mobility scale were separately analyzed following recommendations of a recent study of relational mobility^34^. We calculated each of the relational mobility scores by summing the total score of the individual questions in each category. For sub-population bar plot analysis (Figs. 3, 4 and 5), regression analysis (Tables 2 and 3), and conditional inference tree analysis (Supplementary Figs. S2–S4), relational mobility was kept as this original unbinned continuous score. Histograms of the original scale responses for each type of relational mobility are provided in Supplementary Fig. S1.

The relational mobility scales had moderate internal consistency due to being a more heterogenous psychological construct^31,34^ (Fig. 1A) (Cronbach’s alpha = 0.54 for choosing relational mobility, = 0.62 for meeting relational mobility). The Pearson correlation coefficient between meeting and choosing relational mobility was 0.23. To our knowledge, our study is the largest relational mobility study for a general population sample in Japan (the prior largest study was *N* = 786 of Japanese Facebook users^34^).

#### The number of close friends

The number of close friends was measured by asking subjects to count the number of close friends (outside of the workplace) with whom they can talk about personal matters. We added a note that they should not include their family members or relatives in the count. The possible answers were 0, 1, 2, 3, 4 or 5+. In the subsequent analysis, the answer was used either as a dichotomous variable (0–1 friends/2–4+ friends) (sub-population analysis) or a categorical variable (0, 1, 2, 3, 4, 5+ friends) (regression). In conditional inference trees, 4 and 5+ were binned into 4 + due to lower response frequencies that were present just in the 4 close friends category.

#### Household income

We measured subjects’ income by asking them to indicate their annual household income by choosing one of the six income brackets, ranging from less than two million yen (1) to over ten million yen (6), with an increment of two million yen across brackets. The lowest two income brackets (spanning less than four million yen, approximately $35,000 USD) roughly corresponds to below-middle-class household income in Japan^68^.

A correlation matrix for the primary analysis variables of loneliness, relational mobility (choosing and meeting), number of close friends and income is provided in Supplementary Table S18, with no excessive correlation observed between variables.

#### Additional demographic information

We also collected information on various demographic variables, including their sex, age, marital status, composition of household members, area of residency (prefecture), the characteristics (ruralness) of the area of residency, the length of residency at the current address, subjective assessment of the chance of moving within 12 months, educational attainment, and religiosity. We surveyed other economic conditions as well, including their employment status and recent changes in household financial situation. These variables are discussed further in the Supplementary Information. The full survey and data are provided in Supplementary Data.

### Regression analysis

For the main quantitative analysis, we used the ordinary least squares (OLS) method to study the association between relational mobility, number of close friends, income, and other socioeconomic variables using two models. The first model (Table 2) was as follows:

$$\begin{aligned} y & = \beta_{0} + \beta_{{1}} \cdot NF_{ } + \beta_{{2}} \cdot {\text{ RM - meeting}} + \beta_{{3}} \cdot {\text{ RM - choosing}} + \beta_{{4}} \cdot INC \\ & \;\;\; + \beta_{{5}} \cdot {\text{ GENDER}} + \beta_{{6}} \cdot {\text{ AGE }} + \beta_{{7}} \cdot {\text{ MARRIAGE }} + \gamma \cdot Z + \in \\ \end{aligned}$$

where *y* is the dependent variable (loneliness), *RM* is relational mobility of each type (meeting and choosing), *NF* is number of close friends, *INC* is household income, GENDER is 1 for female and 0 for male, AGE is coded as (1) 20–29, (2) 30–39, (3) 40–49, (4) 50–59, (5) 60–69, and (6) 70 + , MARRIAGE is 1 if married and 0 if not, *Z* is a vector that contains additional secondary regressor variables (Supplementary Table S2), ϵ is an error term, and with the Greek letters being regression coefficients. The continuous (unbinned) loneliness score from the SF-10 scale^47^ was the dependent variable. The sample used in regression was the entire general population survey sample (*N* = 4977).

The second model (Table 3) was mostly the same as the first model, but additionally included interaction terms that further contained mediating variables of gender and age for the two main explanatory variables of number of close friends and relational mobility. The decision to include age and gender interactions was motivated by prior studies of loneliness in Japan which found these variables to have potentially strong associations with loneliness as well^7,18^. The second model is:

$$\begin{aligned} y & = \beta_{0} + \beta_{{1}} \cdot NF + \beta_{{2}} \cdot {\text{ RM - meeting}} + \beta_{{3}} \cdot {\text{ RM - choosing}} + \beta_{{4}} \cdot INC + \beta_{{5}} \cdot {\text{ GENDER}} + \beta_{{6}} \cdot {\text{ AGE}} \\ & \;\; + \beta_{{7}} \cdot NF \cdot {\text{ GENDER}} + \beta_{{8}} \cdot {\text{ RM - meeting}} \cdot {\text{ GENDER}} + \beta_{{9}} \cdot {\text{ RM - choosing}} \cdot {\text{ GENDER}} + \beta_{{{1}0}} \cdot NF \cdot {\text{ AGE }} \\ & \;\; + \beta_{{{11}}} \cdot {\text{ RM - meeting}} \cdot {\text{AGE }} + \beta_{{{12}}} \cdot {\text{ RM - choosing}} \cdot {\text{ AGE }} + \beta_{{{13}}} \cdot {\text{ MARRIAGE }} + \gamma \cdot Z + \in \\ \end{aligned}$$

Each regression was performed on standardized variables (Supplementary Information). After the regression analyses were performed, the Variance Inflation Factor (VIF) was used to check for multicollinearity, with all regressions run found to have < 2 VIF (no multicollinearity).

### Conditional inference tree analysis

Tree-branched models in general recursively split data to produce a tree of ranked sub-populations based on which explanatory variables most influence a certain dependent variable^49^. Typically, when constructing such trees, binary splitting of the data, assessed across all explanatory variables, is done at each step to establish groups that have a between-variation as large, and within-variations as small, as possible. Tree-based methods are especially robust against multicollinearity of explanatory variables and they have no requirement for linearity and normality in explanatory variables as in regression methods. Conditional Inference Trees (CITs), a particularly powerful tree-branched model, use the *party* library in R programming to employ a machine learning algorithm embedded in a conditional inference framework, which use statistical tests such as a *p-*value to determine when further splitting is no longer valid^49,50^. Cross-validation is not required when using CITs because of the statistical tests constraining the branching. Group-level analysis of CITs, i.e. “random forest” analyses of multiple CITs, provide a model that includes all variables that are contributing to explaining variation in the response ranked in order of importance. Thus, using an extensive list of explanatory variables, we present conditional inference tree and forest results for identifying which explanatory variables best define distinct high or low loneliness sub-populations within our semi-representative sample of Japan’s general population. Specifically, we present 1000-tree conditional inference forests that iteratively tested binary splits of explanatory variables to identify the most important variables for creating distinct sub-populations of higher or lower loneliness (Supplementary Fig. S2). As an example, we provided the best fit three-level conditional inference trees that contain one type of relational mobility at a time plus all secondary explanatory variables used in Supplementary Fig. S2 (Supplementary Figs. S3 and S4). See Supplementary Information for an extended description of these methods.

### Sample size sensitivity and robustness checks

Relational mobility is a recently developed psychological construct, and our study is the first to look at within-culture association between individual perceptions of relational mobility and subjective loneliness. Thus, there is a lack of prior literature available to inform power analysis calculations of optimal survey sample sizes in this research topic. However, to our knowledge, our study is the largest relational mobility study for a general population sample in Japan by almost tenfold (the prior largest study was *N* = 786 of Japanese Facebook users^1^ and did not examine within-culture loneliness). We use this advantage to perform several exploratory post-hoc analyses to test the sensitivity of our effect size and statistical significance results to sample size, to inform future work (Supplementary Information Figs. S5–S7). We did not have pilot data to perform a preliminary power analysis and sample size calculation.

### Ethics declaration

This study was approved by the Institutional Review Board (IRB) of RIKEN (Japan), with IRB approval number W2021-020, and was conducted in accordance with both Japanese and international standards of ethical human research. All subjects gave informed consent before participation.

## Supplementary Information

Supplementary Information.

## Data Availability

Upon publication, the original survey and all data used to perform the analyses in this work will be provided in the publicly available Open Science Framework (OSF) directory https://osf.io/sugwe/. Contact ryan.badman113@gmail.com (R.P.B.) for further data requests.
