# Supplementary material for: Perceptions of social rigidity predict loneliness across the Japanese population
Source: Sci Rep. 2022 Sep 27;12:16073. doi: 10.1038/s41598-022-20561-5 (PMC9514195; doi:10.1038/s41598-022-20561-5)
Supplement: Supplementary file 1 — Supplementary Information. [file 41598_2022_20561_MOESM1_ESM.docx]

**SUPPLEMENTARY INFORMATION FOR:**

**Perceptions of Social Rigidity Predict Loneliness Across the Japanese Population**

**Authors:** Ryan P. Badman^1^, Robert Nordström^2^, Michiko Ueda^1,3^ and Rei Akaishi^1^

^1^Center for Brain Science, RIKEN, Wako, Saitama 351-0106, Japan

^2^Graduate School of Political Science, Waseda University, Nishi-Waseda, Shinjuku, Tokyo 169-8050, Japan

^3^Faculty of Political Science, Waseda University, Nishi-Waseda, Shinjuku, Tokyo 169-8050, Japan

**This document contains:**

Extended Methods

Supplementary Figures

Supplementary Tables

**Supplementary** **Methods**

*Explanatory Variables*

Beyond the primary explanatory variables of number of close friends, relational mobility, age, gender, and household income, a larger set of secondary demographics and social psychological explanatory variables were used in both linear regression and conditional inference tree analysis to help isolate out the effects of the primary versus secondary variables (Tables 1-3, Supplementary Tables S1-S5, Supplementary Figures S2-S4). Descriptions of the secondary explanatory variables are provided below, and a full summary table of all explanatory variables used in the main regression analysis (Tables 2 and 3) is presented in Supplementary Table S1.

Respondents who preferred not to answer about their gender or those who chose “other” in the gender category were excluded (*N*=11) due to an unfortunate lack of statistical power in this category. Thus, all the respondents in the analysis were categorized as either female or male. The respondent’s age was categorized into three age groups: 20-29 years old, 30-49 years old, and 50 years and older only in one more fine-grained supplementary regression (Supplementary Tables S4 and S5), and otherwise kept in the original scale (Table 1 and Supplementary Table S1). To capture the household structure, we created four dichotomous variables: *married* for those who are married (regardless of their legal status), *Children in Household* for those living with child(ren) who are less than 18 years old, *Living with (Grand)Parents* for those who are living with either their parents and/or grandparents, and *isSingleParen*t for single parents living with child(ren) younger than 18 years of age.

We categorized the area of residency (Japanese prefecture) into eleven regions according to one of the official Japanese government classifications and included region (*Region*) in regression analyses to capture potential geographic differences in the level of loneliness (Table 1): Hokkaido, Tohoku, North Kanto, South Kanto, Hokuriku, Tokai, Kinki, Chugoku, Shikoku, Kyushu, and Okinawa (Supplementary Data). Hokkaido was used as the reference group in the analysis.

Next, the degree of ruralness of the subjects’ local area of residency was measured from 1 (the most urban area) to 6 (the most rural area). The length of residency at the current address was measured by a monotonically increasing categorical variable that had the options: “Less than one year” (1), “1 year or less than 2 years” (2), “2 years or more and less than 5 years” (3), “5 years or more and less than 10 years” (4), “10 years or more and less than 20 years” (5), or “Over 20 years” (6). The original scale (range 1-6) for was used in the subsequent analysis as the monotonically increasing scale allowed for interpretation of regression coefficient sign. The respondents were also asked to provide their subjective assessment on the likelihood of moving in the next 12 months, another monotonically increasing variable, with options of “Very unlikely” (1), “Unlikely” (2), “I do not know” (3), “There is a slight possibility” (4), and “Very likely” (5). Here again, the original scale (range 1-5) was used in the subsequent analysis as the monotonically increasing scale allowed for interpretation of regression coefficient sign.

We created six mutually exclusive dichotomous variables to measure their education attainment: (1) high school diploma or less (reference group), (2) vocational and technical school, (3) two-year junior college, (4) Bachelor's degree, (5) graduate degree or professional degree, and (6) “other” (unspecified).

Participation in religious activities can affect the level and quality of social connections, and we asked their religiosity which can range from 0 (not religious at all) to 10 (very religious). The scale was entered as it is in the subsequent analysis.

We further take into account whether the respondent was a student by a dichotomous variable *Student Status,* because especially for young people student lifestyles may provide more social support and opportunities to make friends than young professional lifestyles.

We additionally measured whether subjects recently experienced changes in household financial situations by asking them whether they had experienced income loss in the last two years. We created a dichotomous variable (*Recent Worsening of Income*) for respondents who indicated that their income had gone down. Additionally, we calculated whether their income level had changed over the course of their lifespan (*Lifetime Change in Income*). This score was calculated by the difference of two 5-point questions’ scores: *“What is the economic level of your current household?”* minus “*What is the economic level of the family you grew up in?*”. The 5-point scale for each question was organized as “Low” (1) to “High” (5). Thus, a positive difference means the subject had their economic level improve since childhood, a negative difference means the subject had their economic level worsen since childhood.

The subjects’ employment status was measured by a dichotomous variable *Regular Full-time Employment*, which indicates relatively stable salaried employment status.

Last, for a relational stability measure, we summed the scores of the three relational stability questions within the standard scale for this construct^1^. Relational stability as a construct measures subjects’ perceptions of their social environment’s norms about how strongly one should hold on to established relationships.

*ANOVA Tests*

Two-way ANOVA tests were performed on the bar plots in Figure 3 and Figure 4, to test the two-way interactions of relational mobility with separately (1) number of close friends (Supplementary Tables S6-S9) and (2) income (Supplementary Tables S10-S13) respectively. Three-way ANOVA tests were performed on the bar plots in Figure 5 to test the interactions between relational mobility, number of close friends and household income (Supplementary Tables S14-S17). Following ANOVA, Bonferroni-corrected pairwise tests were used to identify whether higher and lower relational mobility sub-groupings had significantly different means within each sub-population.

**Supplementary** **Table S1: Extended summary statistics of survey results (genders combined)**

| **Variable** | **Response** | **N** | **%** | **Loneliness (mean, 95% CI) (0-30 scale)** |
| --- | --- | --- | --- | --- |
| Gender | Male | 2480 | 49.8 | 13.9 (13.8 - 14.1) |
|  | Female | 2497 | 50.2 | 12.8 (12.6 - 13.0) |
|  |  |  |  |  |
| Age groups | 20-29 | 1176 | 23.6 | 13.8 (13.6 - 14.1) |
|  | 30-39 | 748 | 15.0 | 13.9 (13.5 - 14.2) |
|  | 40-49 | 792 | 15.9 | 14.2 (13.8 - 14.5) |
|  | 50-59 | 745 | 15.0 | 13.9 (13.6 - 14.3) |
|  | 60-69 | 794 | 16.0 | 12.6 (12.2 - 12.9) |
|  | 70+ | 722 | 14.5 | 11.4 (11.1 - 11.8) |
|  |  |  |  |  |
| Marital status | Married | 2921 | 58.7 | 12.6 (12.5 - 12.8) |
|  | Not Married | 2056 | 41.3 | 14.4 (14.2 - 14.6) |
|  |  |  |  |  |
| Children | Children in household | 1023 | 20.6 | 13.1 (12.9 - 13.4) |
|  | No children in household | 3954 | 79.4 | 13.4 (13.3 - 13.6) |
|  |  |  |  |  |
| Living with (Grand)Parents | Yes | 1141 | 22.9 | 14.2 (13.9 - 14.5) |
|  | No | 3836 | 77.1 | 13.1 (12.9 - 13.3) |
|  |  |  |  |  |
| Single parent | Yes | 50 | 1 | 13.9 (12.4 - 15.4) |
|  | No | 4927 | 99 | 13.3 (13.2 - 13.5) |
|  |  |  |  |  |
| Degree of Rural | Extremely urban (inside designated city areas) | 1122 | 22.5 | 13.4 (13.1 - 13.7) |
|  | Urban (outside designated city areas) | 431 | 8.7 | 12.9 (12.4 - 13.3) |
|  | Relatively urban | 1201 | 24.1 | 13.1 (12.9 - 13.4) |
|  | Neither urban/rural | 1351 | 27.1 | 13.5 (13.2 - 13.7) |
|  | Relatively rural | 622 | 12.5 | 13.7 (13.3 - 14.1) |
|  | Extremely rural | 250 | 5 | 13.4 (12.7 - 14.0) |
|  |  |  |  |  |
| Length of Current Residence | Less than 1 yr | 240 | 4.8 | 13.9 (13.3 - 14.5) |
|  | More than 1 yr but less than 2 yrs | 248 | 5 | 13.3 (12.7 - 13.9) |
|  | More than 2 yrs but less than 5 yrs | 601 | 12.1 | 13.6 (13.3 - 14.0) |
|  | More than 5 yrs but less than 10 yrs | 490 | 9.8 | 14.2 (13.7 - 14.6) |
|  | More than 10 yrs but less than 20 yrs | 806 | 16.2 | 13.7 (13.4 - 14.0) |
|  | More than 20 yrs | 2592 | 52.1 | 13.0 (12.8 - 13.2) |
|  |  |  |  |  |
| Chance of Moving | Very unlikely | 2702 | 54.3 | 12.8 (12.6 - 13.0) |
|  | Unlikely | 668 | 13.4 | 13.4 (13.1 - 13.7) |
|  | I do not know | 805 | 16.2 | 14.4 (14.1 - 14.7) |
|  | There is a slight possibility | 436 | 8.8 | 14.4 (13.9 - 14.8) |
|  | Very likely | 366 | 7.4 | 14.0 (13.4 - 14.5) |
|  |  |  |  |  |
| Educational attainment | High school diploma or less | 2045 | 41.1 | 13.3 (13.1 - 13.5) |
|  | Vocational/Tech | 268 | 5.4 | 14.0 (13.5 - 14.5) |
|  | Junior College | 1504 | 30.2 | 13.6 (13.4 - 13.9) |
|  | Bachelor's' | 477 | 9.6 | 12.5 (12.0 - 12.9) |
|  | Graduate | 28 | 0.6 | 15.1 (13.6 - 16.6) |
|  | Other | 655 | 13.2 | 13.4 (13.0 - 13.7) |
|  |  |  |  |  |
| Religiousness | 1 (not religious at all) | 1865 | 37.5 | 13.9 (13.7 - 14.1) |
|  | 2 | 579 | 11.6 | 13.6 (13.2 - 14.0) |
|  | 3 | 631 | 12.7 | 13.5 (13.1 - 13.9) |
|  | 4 | 266 | 5.3 | 13.3 (12.8 - 13.8) |
|  | 5 | 746 | 15.0 | 13.0 (12.7 - 13.3) |
|  | 6 | 326 | 6.6 | 13.0 (12.5 - 13.5) |
|  | 7 | 238 | 4.8 | 12.5 (11.8 - 13.1) |
|  | 8 | 152 | 3.1 | 11.6 (10.8 - 12.4) |
|  | 9 | 42 | 0.8 | 12.5 (10.8 - 14.1) |
|  | 10 (very religious) | 132 | 2.7 | 11.1 (10.2 - 12.0) |
|  |  |  |  |  |
| Student | Yes | 375 | 7.5 | 13.6 (13.1 - 14.0) |
|  | No | 4602 | 92.5 | 13.3 (13.2 - 13.5) |
|  |  |  |  |  |
| Number of close friends | 0 | 962 | 19.3 | 16.9 (16.6 - 17.2) |
|  | 1 | 680 | 13.7 | 15.0 (14.7 - 15.3) |
|  | 2 | 1133 | 22.8 | 13.4 (13.2 - 13.7) |
|  | 3 | 854 | 17.2 | 12.0 (11.7 - 12.3) |
|  | 4+ | 1348 | 27.1 | 10.8 (10.5 - 11.0) |
|  |  |  |  |  |
| Income | < 2 million yen | 629 | 12.6 | 14.6 (14.2 - 15.0) |
|  | >= 2 & <4 million yen | 1317 | 26.5 | 13.5 (13.2 - 13.8) |
|  | >= 4 & <6 million yen | 1247 | 25.1 | 13.4 (13.1 - 13.6) |
|  | >=6 & <8 million yen | 845 | 17.0 | 13.0 (12.7 - 13.3) |
|  | >=8 & <10 million yen | 454 | 9.1 | 12.4 (12.0 - 12.8) |
|  | >10 million yen | 485 | 9.7 | 12.9 (12.5 - 13.3) |
|  |  |  |  |  |
| Recent Worsening of Income | Yes | 1234 | 24.8 | 13.2 (13.1 - 13.4) |
|  | No | 3743 | 75.2 | 13.7 (13.5 - 14.0) |
|  |  |  |  |  |
| Lifetime Change in Income | -4 | 15 | 0.3 | 15.1 (11.6 - 18.6) |
|  | -3 | 54 | 1.1 | 15.6 (14.3 - 16.9) |
|  | -2 | 236 | 4.7 | 13.8 (13.1 - 14.5) |
|  | -1 | 729 | 14.6 | 12.9 (12.5 - 13.3) |
|  | 0 | 3113 | 62.5 | 13.5 (13.3 - 13.6) |
|  | 1 | 616 | 12.4 | 12.8 (12.4 - 13.2) |
|  | 2 | 176 | 3.5 | 13.6 (12.8 - 14.3) |
|  | 3 | 32 | 0.6 | 13.9 (11.4 - 16.3) |
|  | 4 | 6 | 0.1 | 10.8 (7.6 - 14.1) |
|  |  |  |  |  |
| Regular Full-time Employment | Yes | 2019 | 40.6 | 13.2 (13.1 - 13.4) |
|  | No | 2958 | 59.4 | 13.5 (13.3 - 13.7) |
|  |  |  |  |  |
| Weekly Free Time | 0 hr | 79 | 1.6 | 15.0 (13.9 - 16.1) |
|  | 1-5 hr | 979 | 19.7 | 13.5 (13.2 - 13.8) |
|  | 5-10 hr | 564 | 11.3 | 13.7 (13.4 - 14.1) |
|  | 10-15 hr | 520 | 10.4 | 13.4 (13.0 - 13.8) |
|  | 15-20 hr | 583 | 11.7 | 12.9 (12.5 - 13.3) |
|  | 20-30 hr | 471 | 9.5 | 12.5 (12.1 - 13.0) |
|  | 30-40 hr | 338 | 6.8 | 12.6 (12.1 - 13.2) |
|  | 40-60 hr | 842 | 16.9 | 13.6 (13.3 - 13.9) |
|  | 60+ hr | 601 | 12.1 | 13.6 (13.2 - 14.1) |
|  |  |  |  |  |
| Regions | Hokkaido | 221 | 4.4 | 13.1 (12.7 - 13.6) |
|  | Tohoku | 638 | 12.8 | 13.4 (12.8 - 14.1) |
|  | North Kanto | 157 | 3.2 | 13.2 (12.2 - 14.2) |
|  | South Kanto | 929 | 18.7 | 13.3 (13.0 - 13.7) |
|  | Hokuriku | 110 | 2.2 | 13.4 (13.0 - 13.7) |
|  | Tokai | 504 | 10.1 | 13.3 (12.5 - 14.1) |
|  | Kinki | 720 | 14.5 | 11.8 (10.4 - 13.2) |
|  | Chugoku | 551 | 11.1 | 13.3 (12.8 - 13.7) |
|  | Shikoku | 508 | 10.2 | 13.4 (13.1 - 13.8) |
|  | Kyushu | 592 | 11.9 | 13.6 (13.2 - 14.0) |
|  | Okinawa | 47 | 0.9 | 13.3 (12.9 - 13.7) |
|  |  |  |  |  |
|  |  |  |  |  |
| Relational Stability | 1, (3-6) | 467 | 9.4 | 14.3 (13.9 - 14.8) |
| *(equal binning of scale range chosen just for summary)* | 2, (7-9) | 1438 | 28.9 | 13.3 (13.1 - 13.5) |
|  | 3, (10-11) | 1268 | 25.5 | 13.0 (12.8 - 13.3) |
|  | 4, (12-14) | 1515 | 30.4 | 13.2 (13.0 - 13.5) |
|  | 5, (15-18) | 289 | 5.8 | 14.1 (13.4 - 14.8) |

**
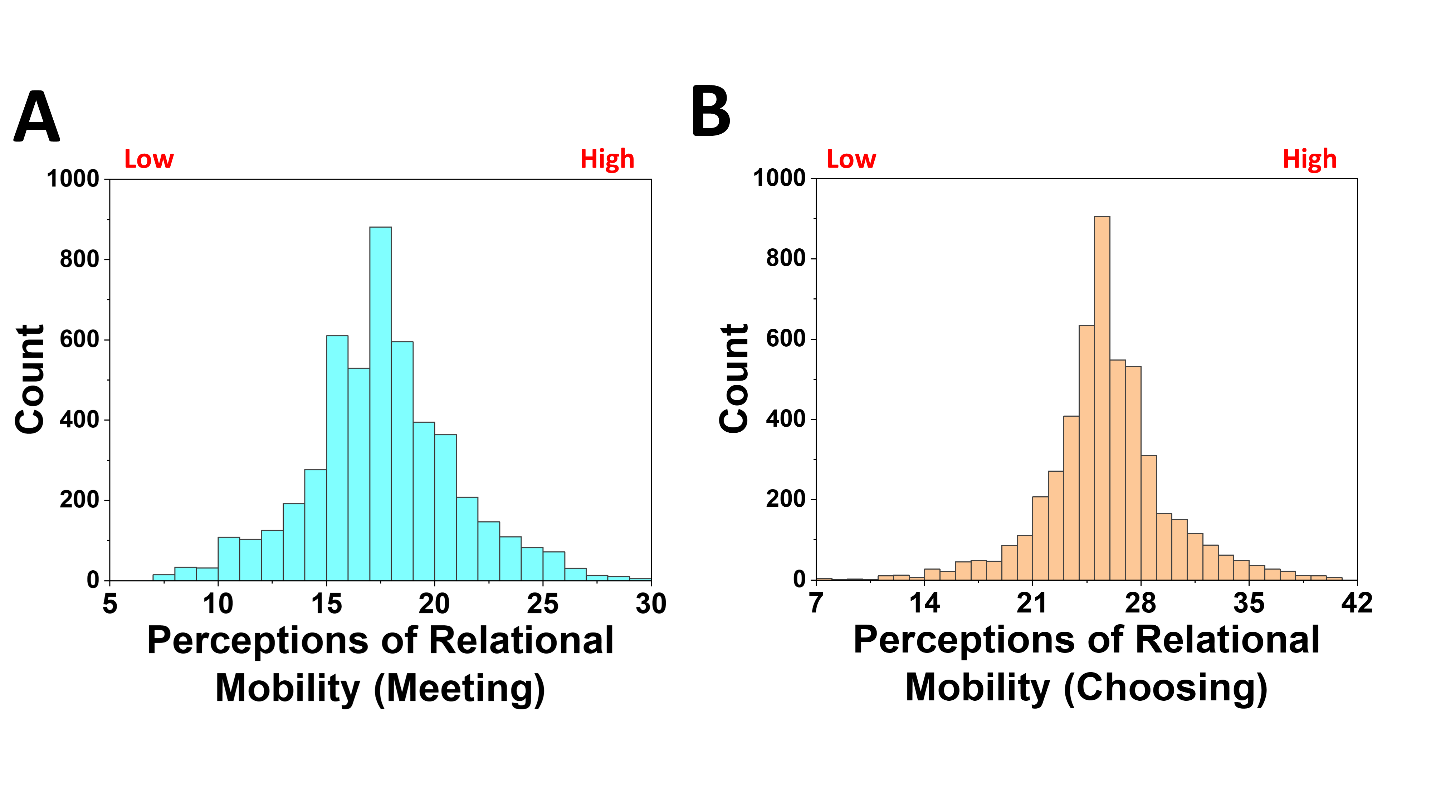
**

**Supplementary** **Figure S1: Histograms of relational mobility scores (standard scales) for all subjects**

For (A) meeting and (B) choosing types of relational mobility scores, histograms are provided for all subjects (*N*=4977) using the original 5-30 and 7-42 scale ranges for (A) and (B) respectively. These continuous standard-scale scores were used in all quantitative analyses.

*Supplementary regression analysis of the factors underlying loneliness*

We next present extended supplementary linear regression results to show the full confounding variable correlations for abbreviated Tables 2 and 3 (Supplementary Tables S2 and S3), and to show separate regression results for different age/gender sub-populations (Supplementary Tables S4 and S5). As in the main text, we designed the regression models with loneliness as the dependent variable, and number of close friends, perceptions of relational mobility, socioeconomic variables, and geographic regional dummy variables as the independent variables. During regression, all variables other than dichotomous -coded (0 or 1) variables were standardized. More specifically, Supplementary Tables S2 and S3 shows the full results of main text Tables 2 and 3. As an additional supplementary regression, we divided subjects into male and female genders and then further into three age groups per gender (20-29 years old, 30-49 years old, and 50+ years old) to further probe robustness of the loneliness and relational mobility correlation and to see how confounding variables affected different ages and genders differently. These age divisions delineated intuitive major life stages to reasonable approximation, given our ten-year interval age scale in the survey (Table 1). Supplementary Tables S4 and S5 show the age- and gender-divided regression results.

Across ages and genders, we found that a higher number of close friends and perceptions of higher relational mobility (both meeting and choosing relational mobility) were by far the strongest and most consistent predictors of reduced loneliness, even when controlling for a long list of diverse socioeconomic variables including income, employment status, age, residential mobility, etc. (Supplementary Table S2-S5). Benefits from both increasing number of close friends and perceptions of higher relational mobility were each approximately the same magnitude as the benefits from being married in reducing loneliness. Additionally, certain age- and gender-dependent trends picked up in the regression analysis may warrant follow-up investigation in future work, at least in the context of Japan (e.g. full-time employment significantly reduces loneliness for middle-aged females, residential mobility increases loneliness and religiousness decreases loneliness for older subjects only, amount of weekly free time affects younger versus older male loneliness differently, single parent males have higher loneliness, etc.). Such trends are beyond the scope of our current work however, in which we tried to focus on the most universal trends across gender.

**Supplementary** **Table S2: Extended OLS regression of factors underlying loneliness in Japanese general population sample**

| **VARIABLES** | **Loneliness** |  |
| --- | --- | --- |
|  |  |  |
|  | *(coefficient)* | *(95% CI)* |
|  |  |  |
| Number of Close Friends | -0.385*** | (-0.409 - -0.360) |
| Relational Mobility (Meeting) | -0.139*** | (-0.164 - -0.114) |
| Relational Mobility (Choosing) | -0.126*** | (-0.152 - -0.099) |
| Income | -0.036** | (-0.063 - -0.008) |
| Gender (Female: 1, Male: 0) | -0.197*** | (-0.249 - -0.145) |
| Age | -0.137*** | (-0.174 - -0.099) |
| Lifetime Change in Income | -0.011 | (-0.036 - 0.013) |
| Recent Worsening of Income | 0.025** | (0.001 - 0.049) |
| Regular Full-time Employment | -0.089*** | (-0.150 - -0.028) |
| Student Status | -0.226*** | (-0.337 - -0.115) |
| Married | -0.227*** | (-0.292 - -0.162) |
| Children in Household | 0.032 | (-0.039 - 0.103) |
| isSingleParent | -0.042 | (-0.288 - 0.203) |
| Weekly Free Time (Hours) | -0.002 | (-0.027 - 0.023) |
| Living with (Grand)Parents | 0.008 | (-0.060 - 0.075) |
| Religiousness | -0.045*** | (-0.070 - -0.020) |
| Relational Stability | -0.001 | (-0.027 - 0.025) |
| Degree of Rural | -0.015 | (-0.041 - 0.011) |
| Length of Current Residence | 0.015 | (-0.013 - 0.043) |
| Chance of Moving | 0.071*** | (0.045 - 0.097) |
| EducationType = 2, Vocational/Tech | -0.082** | (-0.159 - -0.004) |
| EducationType = 3, Junior College | -0.054 | (-0.142 - 0.034) |
| EducationType = 4, Bachelor's' | -0.065** | (-0.125 - -0.006) |
| EducationType = 5, Graduate | 0.039 | (-0.075 - 0.152) |
| EducationType = 6, Other | 0.210 | (-0.104 - 0.523) |
| Region = 2, Tohoku | -0.002 | (-0.131 - 0.127) |
| Region = 3, North Kanto | -0.043 | (-0.215 - 0.130) |
| Region = 4, South Kanto | 0.043 | (-0.081 - 0.167) |
| Region = 5, Hokuriku | -0.016 | (-0.208 - 0.177) |
| Region = 6, Tokai | 0.004 | (-0.128 - 0.137) |
| Region = 7, Kinki | 0.039 | (-0.087 - 0.166) |
| Region = 8, Chugoku | -0.043 | (-0.174 - 0.088) |
| Region = 9, Shikoku | -0.037 | (-0.171 - 0.097) |
| Region = 10, Kyushu | 0.032 | (-0.097 - 0.162) |
| Region = 11, Okinawa | -0.188 | (-0.452 - 0.075) |
| Constant | 0.310*** | (0.178 - 0.442) |
|  |  |  |
| Observations | 4,977 |  |
| R-squared | 0.310 |  |

*p<0.10; **p<0.05; ***p<0.01. Regression coefficients for factors underlying loneliness, with standardized regressors. Negative coefficients correspond to reduced loneliness. 95% confidence intervals are given in parentheses. This is the full regression table for Table 2.

**Supplementary** **Table S3: Extended OLS regression of factors underlying loneliness in Japanese general population sample, with moderator variables of gender and age**

| **VARIABLES** | **Loneliness** |  |
| --- | --- | --- |
|  |  |  |
|  | *(coefficient)* | *(95% CI)* |
|  |  |  |
| Number of Close Friends | -0.349*** | (-0.382 - -0.316) |
| Relational Mobility (Meeting) | -0.128*** | (-0.166 - -0.091) |
| Relational Mobility (Choosing) | -0.084*** | (-0.123 - -0.045) |
| Income | -0.037*** | (-0.064 - -0.010) |
| Gender (Female: 1, Male: 0) | -0.197*** | (-0.248 - -0.145) |
| Age | -0.134*** | (-0.171 - -0.096) |
| *Interaction Term*: Number of Close Friends & Gender | -0.077*** | (-0.125 - -0.029) |
| *Interaction Term*: Number of Close Friends & Age | -0.046*** | (-0.070 - -0.022) |
| *Interaction Term*: Relational Mobility (Meeting) & Gender | -0.014 | (-0.063 - 0.035) |
| *Interaction Term*: Relational Mobility (Meeting) & Age | -0.015 | (-0.040 - 0.009) |
| *Interaction Term*: Relational Mobility (Choosing) & Gender | -0.072*** | (-0.121 - -0.023) |
| *Interaction Term*: Relational Mobility (Choosing) & Age | -0.016 | (-0.040 - 0.008) |
| Married | -0.233*** | (-0.298 - -0.169) |
| Lifetime Change in Income | -0.009 | (-0.033 - 0.015) |
| Recent Worsening of Income | 0.023* | (-0.001 - 0.047) |
| Regular Full-time Employment | -0.093*** | (-0.154 - -0.032) |
| Student Status | -0.246*** | (-0.356 - -0.135) |
| Children in Household | 0.034 | (-0.036 - 0.105) |
| isSingleParent | -0.048 | (-0.293 - 0.196) |
| Weekly Free Time (Hours) | -0.002 | (-0.027 - 0.023) |
| Living with (Grand)Parents | 0.003 | (-0.064 - 0.070) |
| Religiousness | -0.040*** | (-0.065 - -0.015) |
| Relational Stability | -0.003 | (-0.029 - 0.023) |
| Degree of Rural | -0.016 | (-0.042 - 0.010) |
| Length of Current Residence | 0.017 | (-0.011 - 0.045) |
| Chance of Moving | 0.070*** | (0.044 - 0.096) |
| EducationType = 2, Vocational/Tech | -0.082** | (-0.160 - -0.005) |
| EducationType = 3, Junior College | -0.053 | (-0.140 - 0.035) |
| EducationType = 4, Bachelor's' | -0.065** | (-0.125 - -0.005) |
| EducationType = 5, Graduate | 0.041 | (-0.072 - 0.154) |
| EducationType = 6, Other | 0.211 | (-0.101 - 0.523) |
| Region = 2, Tohoku | 0.003 | (-0.126 - 0.132) |
| Region = 3, North Kanto | -0.045 | (-0.217 - 0.127) |
| Region = 4, South Kanto | 0.048 | (-0.075 - 0.172) |
| Region = 5, Hokuriku | -0.009 | (-0.201 - 0.183) |
| Region = 6, Tokai | 0.010 | (-0.122 - 0.142) |
| Region = 7, Kinki | 0.042 | (-0.084 - 0.168) |
| Region = 8, Chugoku | -0.041 | (-0.172 - 0.090) |
| Region = 9, Shikoku | -0.031 | (-0.164 - 0.103) |
| Region = 10, Kyushu | 0.039 | (-0.090 - 0.168) |
| Region = 11, Okinawa | -0.192 | (-0.454 - 0.071) |
| Constant | 0.317*** | (0.186 - 0.449) |
|  |  |  |
| Observations | 4,977 |  |
| R-squared | 0.316 |  |

*p<0.10; **p<0.05; ***p<0.01. Regression coefficients for factors underlying loneliness, with standardized regressors. Negative coefficients correspond to reduced loneliness. 95% confidence intervals are given in parentheses. This is the full regression table for Table 3.

**Supplementary** **Table S4: OLS regression of factors underlying male loneliness for three age groups**

|  | (1) | (2) | (3) |
| --- | --- | --- | --- |
| **VARIABLES** | **Loneliness**  **(20-29 yo)** | **Loneliness**  **(30-49 yo)** | **Loneliness (M3)**  **(50+ yo)** |
|  |  |  |  |
| Number of Close Friends | -0.288*** | -0.355*** | -0.420*** |
| *(95% confidence intervals)* | (-0.367 - -0.208) | (-0.416 - -0.294) | (-0.470 - -0.371) |
| Relational Mobility (Meeting) | -0.131** | -0.168*** | -0.107*** |
|  | (-0.230 - -0.031) | (-0.236 - -0.101) | (-0.163 - -0.052) |
| Relational Mobility (Choosing) | -0.020 | -0.126*** | -0.111*** |
|  | (-0.122 - 0.083) | (-0.203 - -0.050) | (-0.170 - -0.052) |
| Income | -0.064 | -0.082** | -0.055* |
|  | (-0.149 - 0.021) | (-0.159 - -0.004) | (-0.114 - 0.004) |
| Lifetime Change in Income | 0.078 | 0.015 | -0.075*** |
|  | (-0.023 - 0.179) | (-0.051 - 0.082) | (-0.122 - -0.028) |
| Recent Worsening of Income | 0.010 | 0.001 | 0.005 |
|  | (-0.071 - 0.091) | (-0.055 - 0.057) | (-0.049 - 0.058) |
| Regular Full-time Employment | -0.108 | -0.100 | 0.033 |
|  | (-0.352 - 0.135) | (-0.277 - 0.076) | (-0.083 - 0.149) |
| Student Status | -0.180 | 0.275 | N/A |
|  | (-0.436 - 0.075) | (-0.924 - 1.473) | N/A |
| Married | -0.182 | -0.259*** | -0.370*** |
|  | (-0.472 - 0.108) | (-0.443 - -0.075) | (-0.501 - -0.239) |
| Children in Household | -0.100 | 0.018 | 0.179* |
|  | (-0.465 - 0.266) | (-0.148 - 0.184) | (-0.000 - 0.359) |
| isSingleParent | N/A | 0.738** | 0.142 |
|  | N/A | (0.108 - 1.367) | (-1.475 - 1.759) |
| Weekly Free Time (Hours) | 0.122*** | 0.045 | -0.047* |
|  | (0.037 - 0.207) | (-0.029 - 0.119) | (-0.097 - 0.002) |
| Living with (Grand)Parents | 0.013 | 0.009 | -0.136* |
|  | (-0.200 - 0.226) | (-0.167 - 0.185) | (-0.278 - 0.006) |
| Religiousness | 0.027 | -0.045 | -0.076*** |
|  | (-0.059 - 0.113) | (-0.110 - 0.020) | (-0.125 - -0.026) |
| Relational Stability | -0.015 | -0.041 | -0.006 |
|  | (-0.093 - 0.063) | (-0.108 - 0.027) | (-0.063 - 0.051) |
| Degree of Rural | -0.006 | 0.038 | -0.017 |
|  | (-0.091 - 0.079) | (-0.029 - 0.106) | (-0.069 - 0.034) |
| Length of Current Residence | 0.050 | 0.018 | -0.007 |
|  | (-0.032 - 0.131) | (-0.049 - 0.086) | (-0.082 - 0.067) |
| Chance of Moving | 0.008 | 0.060* | 0.103*** |
|  | (-0.067 - 0.083) | (-0.002 - 0.121) | (0.040 - 0.167) |
| EducationType = 2, Vocational/Tech | 0.027 | -0.094 | 0.007 |
|  | (-0.254 - 0.308) | (-0.296 - 0.109) | (-0.173 - 0.187) |
| EducationType = 3, Junior College | 0.447 | 0.349 | -0.183 |
|  | (-0.198 - 1.092) | (-0.094 - 0.792) | (-0.486 - 0.121) |
| EducationType = 4, Bachelor's' | -0.013 | -0.062 | -0.067 |
|  | (-0.215 - 0.190) | (-0.219 - 0.095) | (-0.180 - 0.046) |
| EducationType = 5, Graduate | 0.117 | 0.060 | 0.072 |
|  | (-0.197 - 0.431) | (-0.178 - 0.299) | (-0.141 - 0.286) |
| EducationType = 6, Other | 0.126 | 0.100 | 0.207 |
|  | (-0.656 - 0.908) | (-0.746 - 0.946) | (-0.593 - 1.007) |
| Region = 2, Tohoku | 0.020 | -0.165 | 0.002 |
|  | (-0.405 - 0.444) | (-0.512 - 0.182) | (-0.260 - 0.263) |
| Region = 3, North Kanto | -0.039 | 0.202 | -0.027 |
|  | (-0.593 - 0.515) | (-0.258 - 0.662) | (-0.367 - 0.313) |
| Region = 4, South Kanto | 0.261 | 0.197 | -0.096 |
|  | (-0.152 - 0.674) | (-0.126 - 0.521) | (-0.346 - 0.154) |
| Region = 5, Hokuriku | -0.081 | -0.150 | 0.021 |
|  | (-0.834 - 0.673) | (-0.597 - 0.296) | (-0.372 - 0.413) |
| Region = 6, Tokai | 0.027 | 0.109 | -0.003 |
|  | (-0.409 - 0.462) | (-0.248 - 0.466) | (-0.271 - 0.264) |
| Region = 7, Kinki | 0.131 | 0.056 | 0.033 |
|  | (-0.289 - 0.552) | (-0.280 - 0.392) | (-0.223 - 0.289) |
| Region = 8, Chugoku | 0.196 | 0.110 | -0.150 |
|  | (-0.235 - 0.626) | (-0.243 - 0.463) | (-0.417 - 0.116) |
| Region = 9, Shikoku | 0.199 | -0.132 | -0.054 |
|  | (-0.237 - 0.634) | (-0.490 - 0.226) | (-0.327 - 0.218) |
| Region = 10, Kyushu | 0.226 | 0.152 | 0.031 |
|  | (-0.203 - 0.655) | (-0.194 - 0.497) | (-0.230 - 0.291) |
| Region = 11, Okinawa | -0.387 | 0.043 | -0.450 |
|  | (-1.141 - 0.367) | (-0.569 - 0.655) | (-1.054 - 0.154) |
|  |  |  |  |
| Observations | 590 | 775 | 1,115 |
| R-squared | 0.181 | 0.323 | 0.380 |

*p<0.10; **p<0.05; ***p<0.01. Regression coefficients for factors underlying loneliness for males of different ages: 20-29, 30-49, 50+, with standardized regressors. Negative coefficients correspond to reduced loneliness.

**Supplementary** **Table S5: OLS regression of factors underlying female loneliness for three age groups**

|  | (1) | (2) | (3) |
| --- | --- | --- | --- |
| **VARIABLES** | **Loneliness**  **(20-29 yo)** | **Loneliness**  **(30-49 yo)** | **Loneliness**  **(50+ yo)** |
|  |  |  |  |
| Number of Close Friends | -0.426*** | -0.335*** | -0.427*** |
| *(95% confidence intervals)* | (-0.507 - -0.344) | (-0.405 - -0.265) | (-0.478 - -0.376) |
| Relational Mobility (Meeting) | -0.123*** | -0.103*** | -0.172*** |
|  | (-0.197 - -0.048) | (-0.166 - -0.040) | (-0.221 - -0.122) |
| Relational Mobility (Choosing) | -0.134*** | -0.125*** | -0.143*** |
|  | (-0.212 - -0.056) | (-0.193 - -0.057) | (-0.194 - -0.091) |
| Income | -0.025 | -0.061 | 0.003 |
|  | (-0.102 - 0.051) | (-0.144 - 0.021) | (-0.053 - 0.058) |
| Lifetime Change in Income | -0.048 | 0.008 | 0.029 |
|  | (-0.133 - 0.037) | (-0.053 - 0.069) | (-0.018 - 0.076) |
| Recent Worsening of Income | 0.023 | 0.053* | 0.017 |
|  | (-0.047 - 0.093) | (-0.009 - 0.116) | (-0.040 - 0.073) |
| Regular Full-time Employment | -0.060 | -0.212*** | 0.044 |
|  | (-0.262 - 0.142) | (-0.357 - -0.067) | (-0.107 - 0.196) |
| Student Status | -0.107 | -0.404 | -0.329 |
|  | (-0.341 - 0.128) | (-1.291 - 0.482) | (-1.933 - 1.275) |
| Married | -0.213 | -0.343*** | -0.186*** |
|  | (-0.481 - 0.055) | (-0.549 - -0.137) | (-0.300 - -0.072) |
| Children in Household | 0.059 | 0.037 | -0.030 |
|  | (-0.245 - 0.364) | (-0.141 - 0.215) | (-0.224 - 0.164) |
| isSingleParent | 0.132 | -0.323* | -0.225 |
|  | (-0.714 - 0.979) | (-0.703 - 0.056) | (-0.905 - 0.455) |
| Weekly Free Time (Hours) | 0.006 | 0.019 | -0.051** |
|  | (-0.077 - 0.089) | (-0.055 - 0.092) | (-0.098 - -0.004) |
| Living with (Grand)Parents | -0.035 | 0.057 | 0.030 |
|  | (-0.249 - 0.179) | (-0.126 - 0.240) | (-0.130 - 0.191) |
| Religiousness | 0.064 | -0.050 | -0.078*** |
|  | (-0.030 - 0.158) | (-0.123 - 0.022) | (-0.123 - -0.033) |
| Relational Stability | 0.001 | 0.023 | 0.040 |
|  | (-0.080 - 0.082) | (-0.050 - 0.095) | (-0.015 - 0.096) |
| Degree of Rural | -0.012 | -0.006 | -0.032 |
|  | (-0.095 - 0.070) | (-0.084 - 0.071) | (-0.086 - 0.022) |
| Length of Current Residence | -0.018 | 0.030 | -0.067* |
|  | (-0.098 - 0.063) | (-0.042 - 0.102) | (-0.140 - 0.007) |
| Chance of Moving | 0.047 | 0.089** | 0.150*** |
|  | (-0.018 - 0.112) | (0.019 - 0.158) | (0.086 - 0.214) |
| EducationType = 2, Vocational/Tech | 0.008 | -0.140 | -0.108 |
|  | (-0.233 - 0.249) | (-0.338 - 0.059) | (-0.256 - 0.040) |
| EducationType = 3, Junior College | -0.092 | -0.095 | -0.039 |
|  | (-0.403 - 0.219) | (-0.297 - 0.106) | (-0.164 - 0.087) |
| EducationType = 4, Bachelor's' | -0.008 | -0.064 | -0.013 |
|  | (-0.202 - 0.187) | (-0.246 - 0.117) | (-0.146 - 0.120) |
| EducationType = 5, Graduate | 0.099 | 0.025 | -0.067 |
|  | (-0.403 - 0.601) | (-0.281 - 0.331) | (-0.558 - 0.424) |
| EducationType = 6, Other | -0.744 | 0.075 | 0.711* |
|  | (-1.996 - 0.508) | (-0.603 - 0.753) | (-0.006 - 1.428) |
| Region = 2, Tohoku | -0.120 | -0.150 | 0.204 |
|  | (-0.527 - 0.287) | (-0.508 - 0.208) | (-0.053 - 0.461) |
| Region = 3, North Kanto | -0.095 | -0.685*** | 0.205 |
|  | (-0.647 - 0.456) | (-1.152 - -0.218) | (-0.162 - 0.572) |
| Region = 4, South Kanto | -0.093 | -0.130 | 0.128 |
|  | (-0.497 - 0.312) | (-0.471 - 0.211) | (-0.119 - 0.375) |
| Region = 5, Hokuriku | 0.120 | -0.432 | 0.199 |
|  | (-0.499 - 0.738) | (-0.948 - 0.084) | (-0.209 - 0.607) |
| Region = 6, Tokai | 0.010 | -0.286 | 0.170 |
|  | (-0.414 - 0.433) | (-0.655 - 0.083) | (-0.091 - 0.431) |
| Region = 7, Kinki | -0.188 | -0.069 | 0.173 |
|  | (-0.590 - 0.215) | (-0.419 - 0.281) | (-0.079 - 0.424) |
| Region = 8, Chugoku | -0.280 | -0.327* | 0.186 |
|  | (-0.691 - 0.131) | (-0.693 - 0.039) | (-0.075 - 0.447) |
| Region = 9, Shikoku | -0.236 | -0.175 | 0.111 |
|  | (-0.655 - 0.183) | (-0.550 - 0.200) | (-0.157 - 0.380) |
| Region = 10, Kyushu | -0.101 | -0.341* | 0.236* |
|  | (-0.511 - 0.309) | (-0.699 - 0.016) | (-0.021 - 0.493) |
| Region = 11, Okinawa | -0.253 | -0.372 | 0.031 |
|  | (-1.199 - 0.693) | (-1.057 - 0.314) | (-0.548 - 0.609) |
|  |  |  |  |
| Observations | 586 | 765 | 1,146 |
| R-squared | 0.271 | 0.267 | 0.374 |

*p<0.10; **p<0.05; ***p<0.01. Regression coefficients for factors underlying loneliness for females of different ages: 20-29, 30-49, 50+, with standardized regressors. Negative coefficients correspond to reduced loneliness.

**Supplementary Table S6: ANOVA results for Figure 3A, with number of close friends and relational mobility (meeting) for females**

**
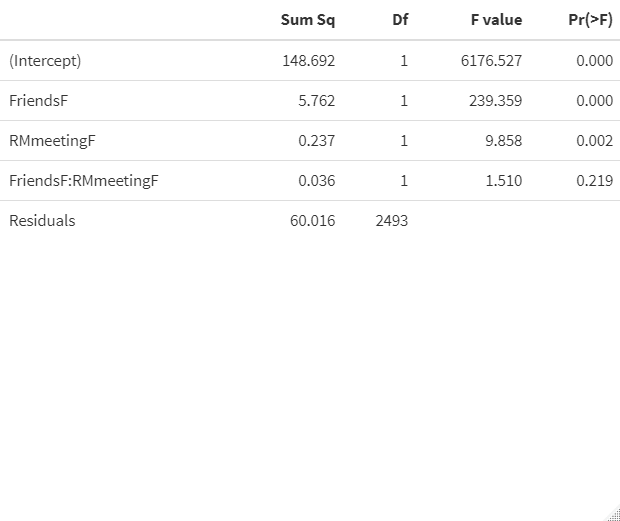
**

**Supplementary** **Table S7: ANOVA results for Figure 3B, with number of close friends and relational mobility (choosing) for females**

**
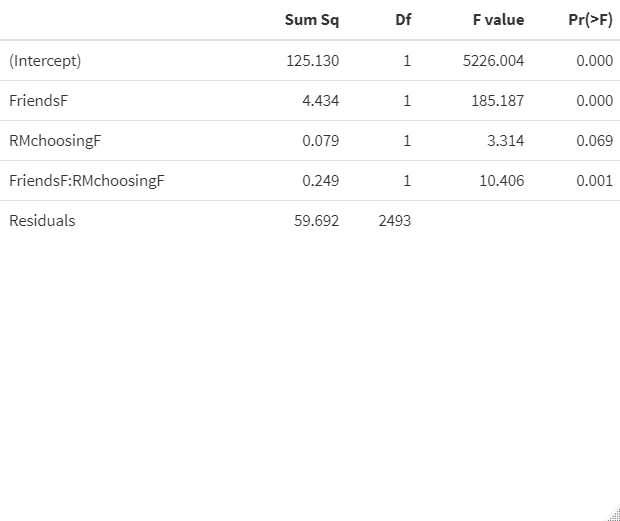
**

**Supplementary** **Table S8: ANOVA results for Figure 3C, with number of close friends and relational mobility (meeting) for males**


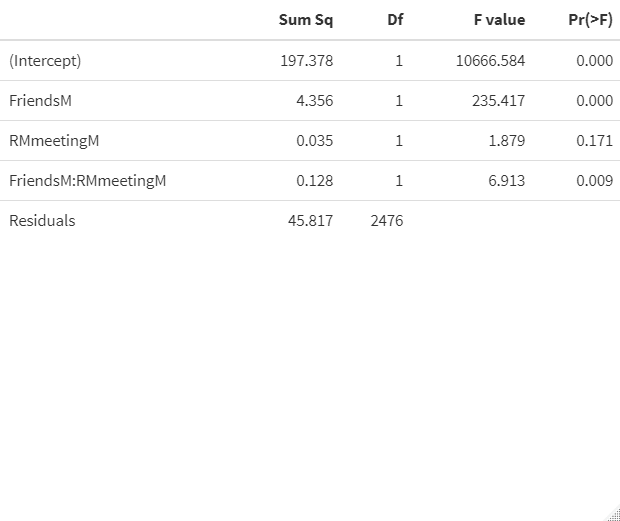


**Supplementary** **Table S9: ANOVA results for Figure 3D, with number of close friends and relational mobility (choosing) for males**
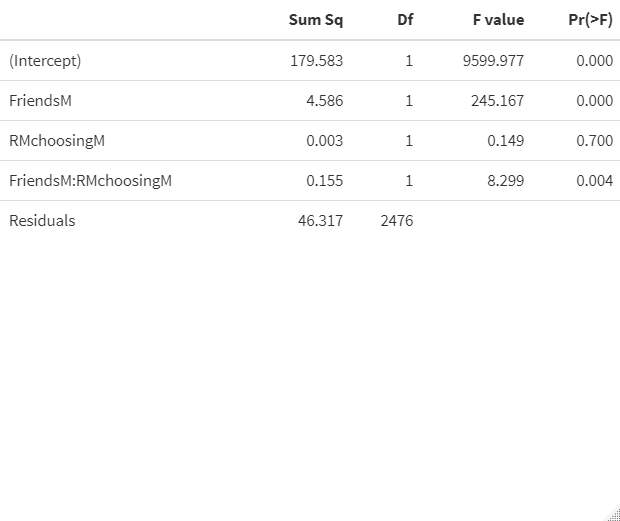


**Supplementary** **Table S10: ANOVA results for Figure 4A, with income and relational mobility (meeting) for females**


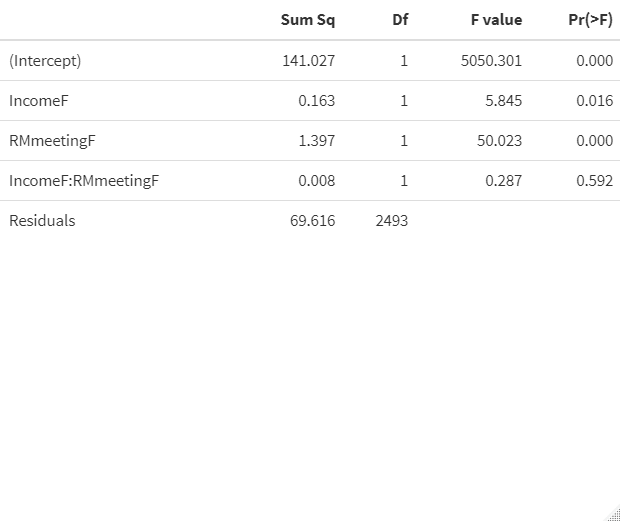


**Supplementary** **Table S11: ANOVA results for Figure 4B, with income and relational mobility (choosing) for females**


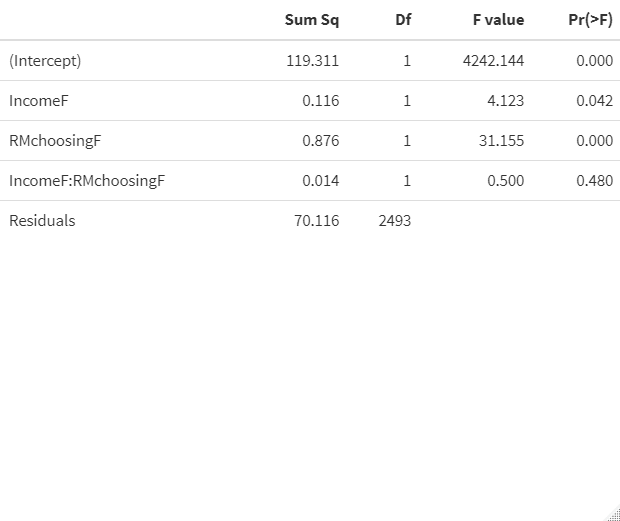


**Supplementary** **Table S12: ANOVA results for Figure 4C, with income and relational mobility (meeting) for males**


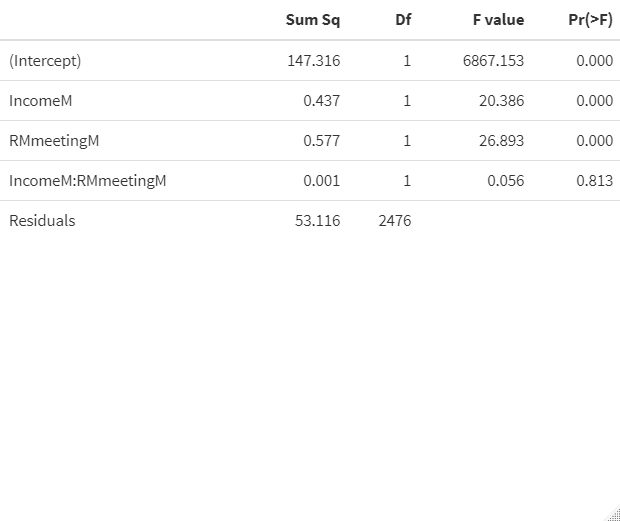


**Supplementary** **Table S13: ANOVA results for Figure 4D, with income and relational mobility (choosing) for males**


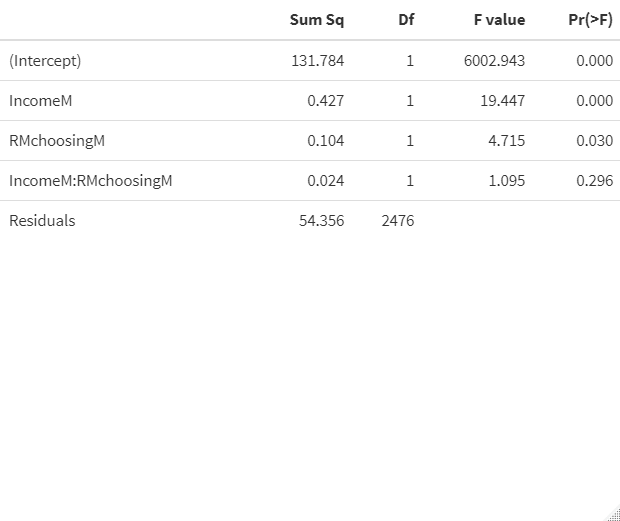


**Supplementary** **Table S14: ANOVA results for Figure 5A, with number of close friends, income and relational mobility (meeting) for females
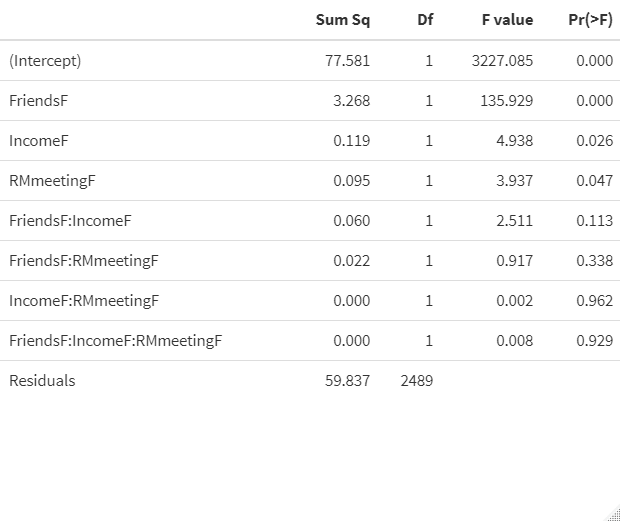
**

**Supplementary** **Table S15: ANOVA results for Figure 5B, with number of close friends, income and relational mobility (choosing) for females
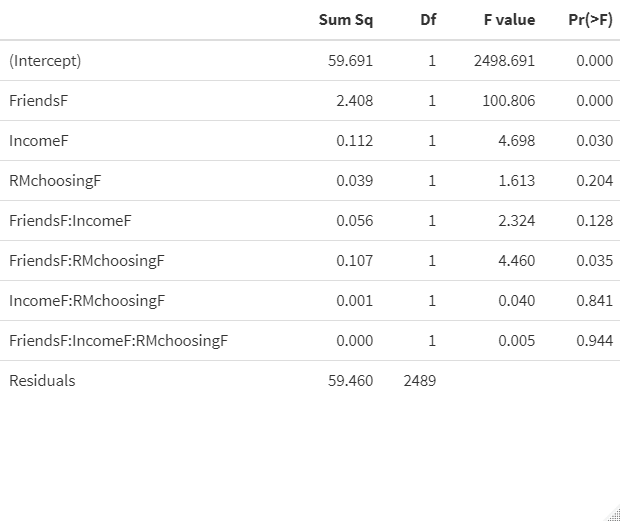
**

**Supplementary** **Table S16: ANOVA results for Figure 5C, with number of close friends, income and relational mobility (meeting) for males**


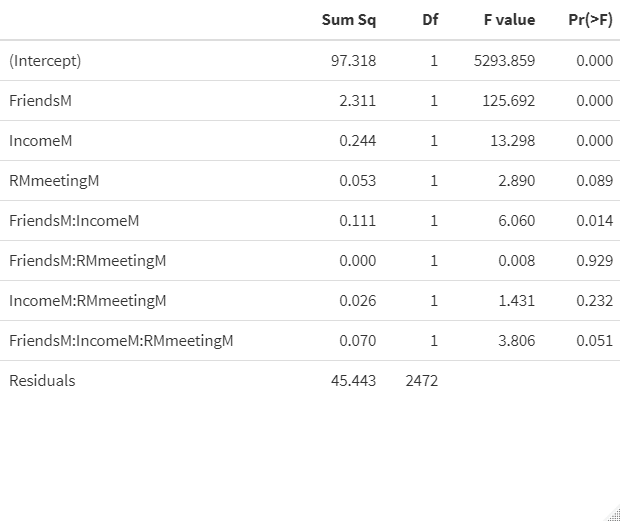


**Supplementary** **Table S17: ANOVA results for Figure 5D, with number of close friends, income and relational mobility (choosing) for males
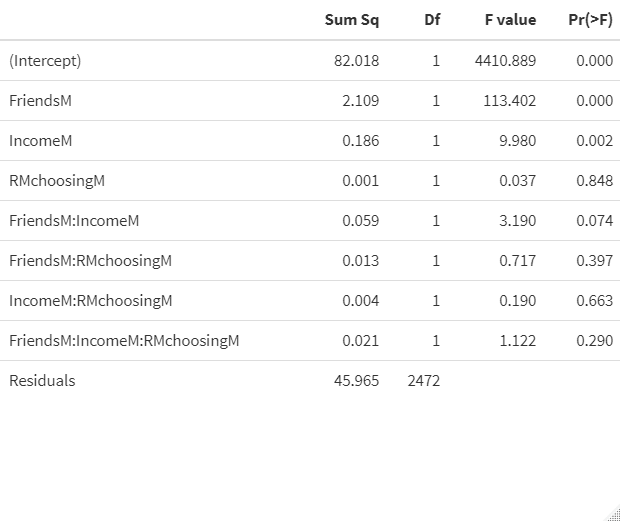
**

**Supplementary** **Table S18: Pearson correlation coefficients for primary analysis variables for all subjects**

|  |  |  |  |  |  |  |
| --- | --- | --- | --- | --- | --- | --- |
|  |  |  |  |  |  |  |
|  | Loneliness | Relational Mobility (Meeting) | Relational Mobility (Choosing) | Number of Close Friends | Income | Age |
| Loneliness | 1.00 |  |  |  |  |  |
| Relational Mobility (Meeting) | -0.26^***^ | 1.00 |  |  |  |  |
| Relational Mobility (Choosing) | -0.22^***^ | 0.23^***^ | 1.00 |  |  |  |
| Number of Close Friends | -0.45^***^ | 0.22^***^ | 0.14^***^ | 1.00 |  |  |
| Income | -0.10^***^ | 0.10^***^ | -0.03 | 0.12^***^ | 1.00 |  |
| Age | -0.15^***^ | -0.07^***^ | 0.01 | -0.03 | -0.10^***^ | 1.00 |

*Conditional inference trees*

Tree-branched models in general recursively divide data to produce a tree of ranked sub-populations based on explanatory variables which most influence a certain dependent variable^2^. Typically when constructing such trees, binary splitting of the data, assessed across all explanatory variables, is done at each step to establish groups that have a between-variation as large, and within-variations as small, as possible^2^. Tree-based methods are especially robust against multicollinearity of explanatory variables and they have no requirement for linearity and normality in explanatory variables as in regression methods. Traditional tree-branched models such as classification and regression trees have the downside that they can easily be overfit, are prone to variable selection bias, and require somewhat subjective and manual pruning methods^2,3^. In contrast, the newer tree method of Conditional Inference Trees (CITs) that use the *party* library in R programming^2^ resolves these downsides as CITs still perform the core binary recursive partitioning, but instead use a machine learning algorithm embedded in a conditional inference framework. While, traditional classification and regression trees continue splitting until no further splits are possible, in contrast CITs use stopping criterion determined by statistical testing, i.e. an a priori *p-*value, to statistically determine when further splitting is no longer valid^2^. Additionally, cross-validation is necessary to prune traditional classification and regression trees, but is not required when using CITs because of the statistical tests constraining the branching. Thus, CITs are significantly more robust against over-fitting and are less biased with regard to the types of explanatory variables used than traditional tree methods^2,3^. Then, because as a remaining potential downside, individual CITs do potentially risk choosing a variable that is acting as a proxy for several other variables that are modulating the dependent variable, random forest analyses of multiple CITs help overcome this risk. Forests provide a model that includes all variables that are contributing to explaining variation in the response ranked in order of importance, so that any such proxy variable should all appear high in the importance ranking^3^.

Thus, using an extensive list of explanatory variables, we present conditional inference tree and forest results for identifying which explanatory variables best define distinct high or low loneliness sub-populations within our semi-representative sample of Japan’s general population. Specifically, we present 1000-tree conditional inference forests that iteratively tested binary splits of explanatory variables to identify the most important variables for creating distinct sub-populations of higher or lower loneliness (Supplementary Figure S2). We then present the best fit three-level conditional inference trees that contain one type of relational mobility at a time plus all secondary explanatory variables used in Supplementary Figure S2 (Supplementary Figure S3 and S4).


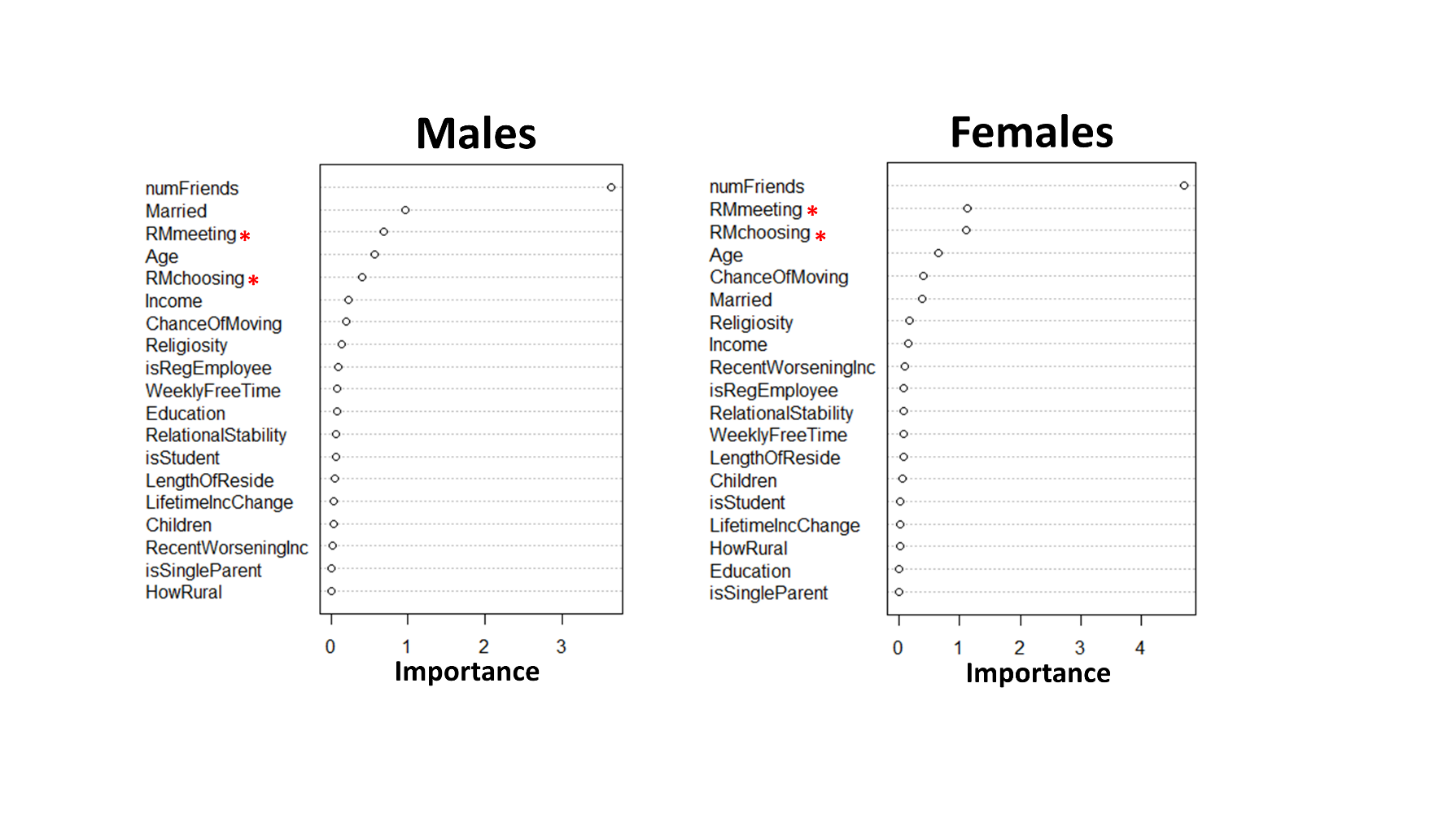


**Supplementary** **Figure S2: Random forest results of importance of each explanatory variable for identifying sub-populations with significantly different loneliness in Japan**

Results of 1000-tree conditional inference forests for males (left) and females (right), plotting the importance of each variable for influencing loneliness clustering for all explanatory variables considered. The importance measure for conditional inference trees is related to how much the variable contributes to maximizing the ratio of between group variance to the within-group variance of sub-populations with statistically different levels of loneliness, and does not consider sign (positive or negative effects on loneliness are treated the same). The two types of relational mobility, choosing and meeting, are marked in red (*), and are among the top ranked explanatory variables for clustering sub-populations of significantly different loneliness distributions in our data sample.


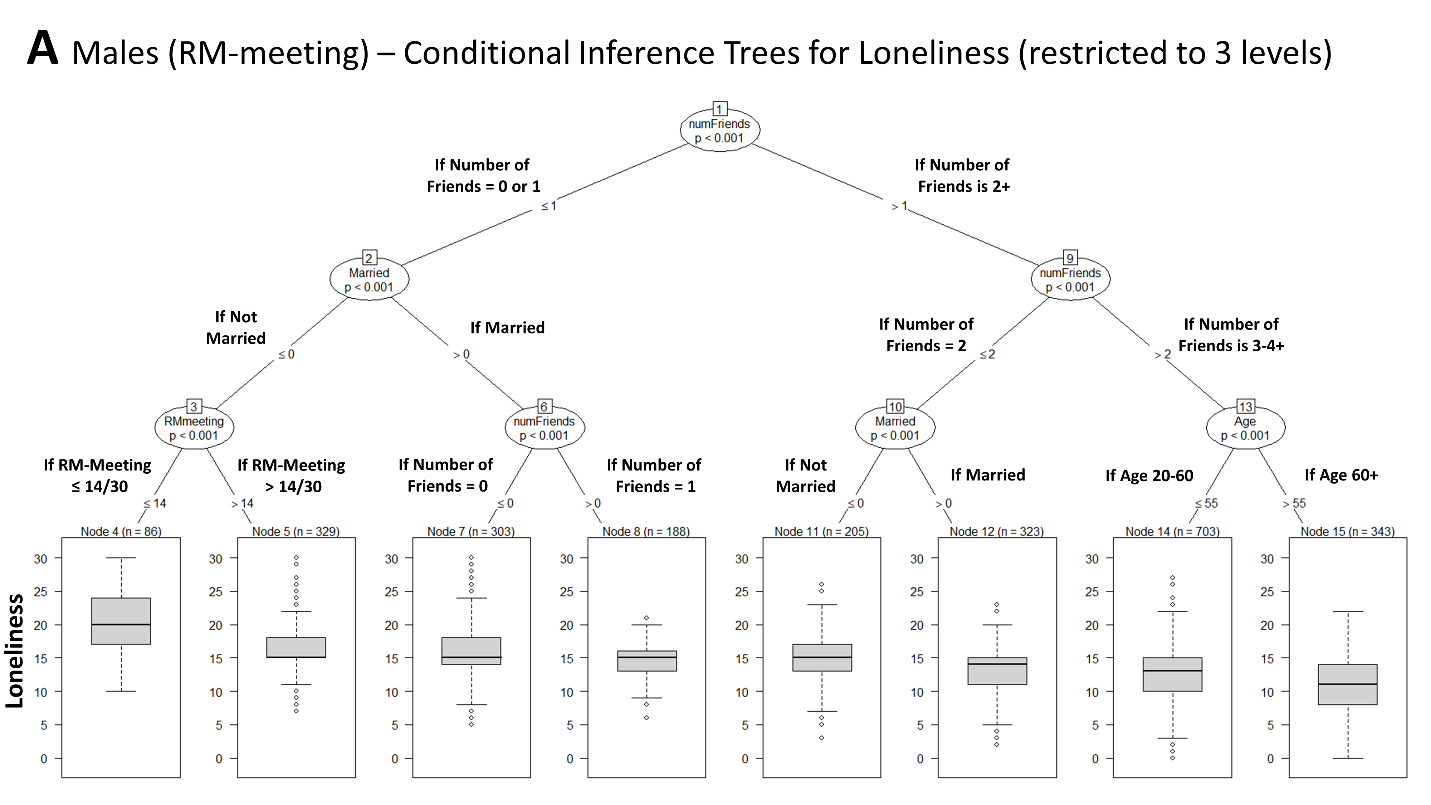

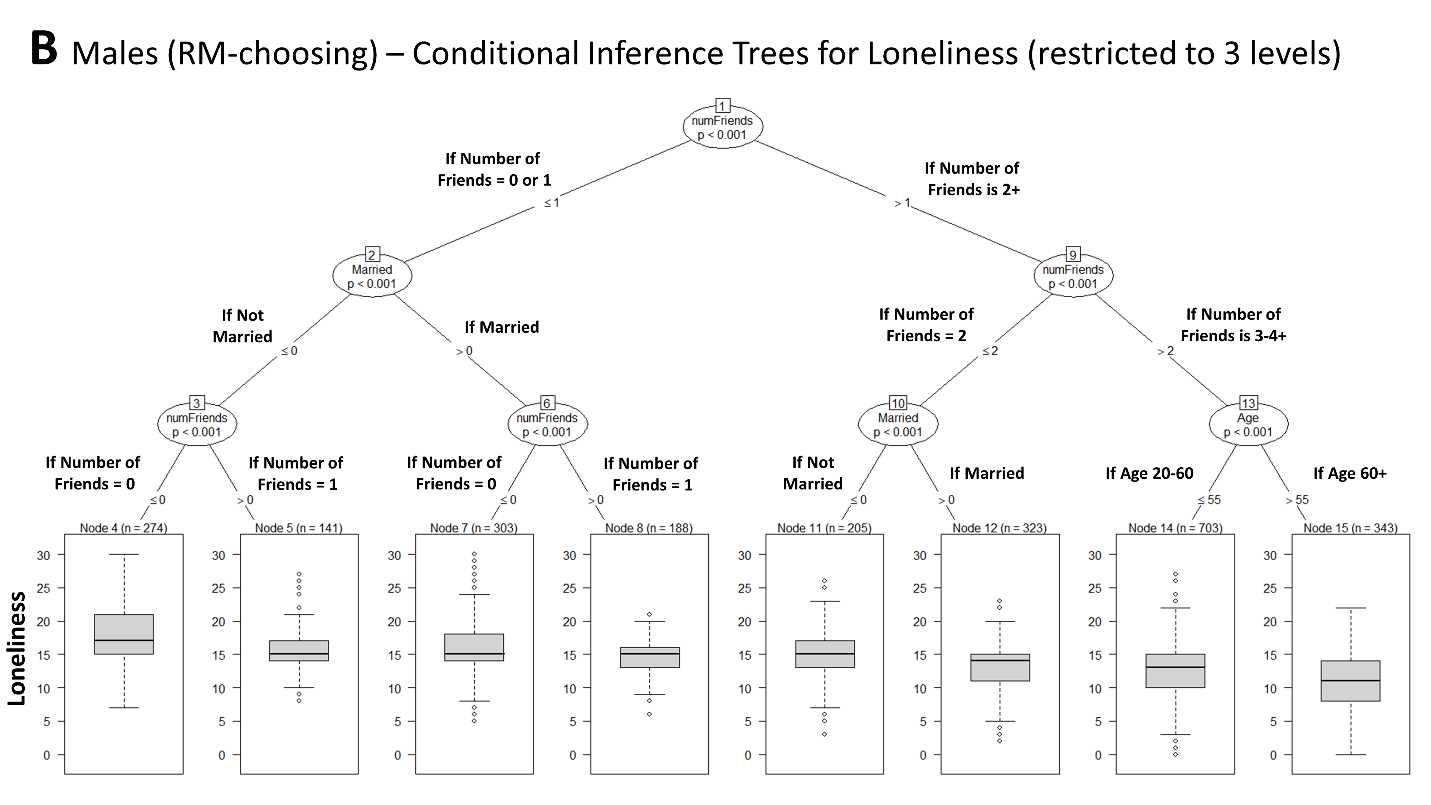


**Supplementary** **Figure S3: Conditional inference tree of major factors that predict loneliness in male sub-populations in Japan**

For male subjects only: the top three levels in importance of the best conditional inference tree for explanatory variables that maximize the ratio of the between-group variance to within-group variance for loneliness distributions within sub-populations in our general population sample. The highest branch has the most important variable for splitting, and then lower branches have descending importance. All explanatory variables that are included in Supplementary Figure S2 were input into the tree algorithm, other than in (A) choosing relational mobility was excluded as this tree just tested meeting relational mobility, and other than in (B) meeting relational mobility was excluded as this tree just tested choosing relational mobility.


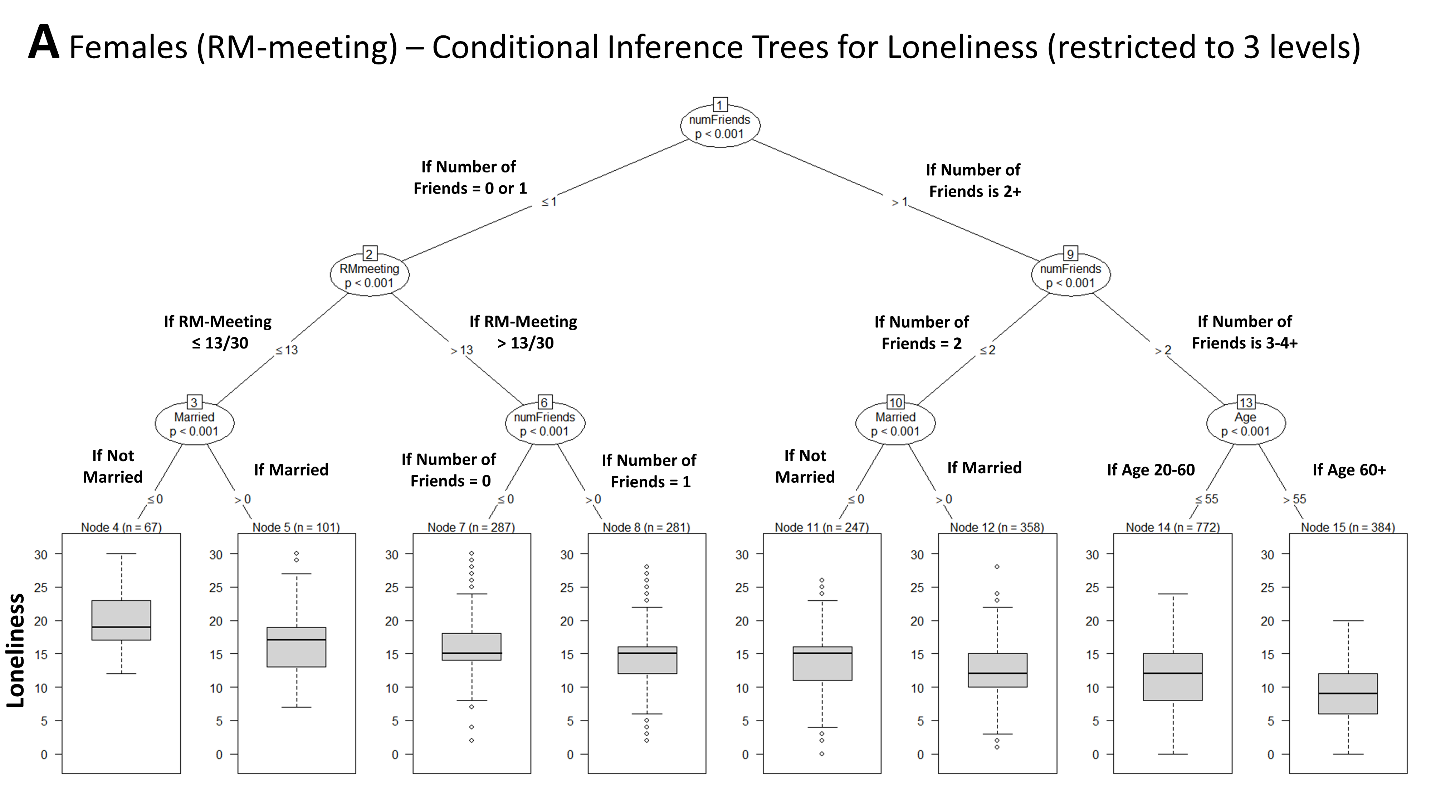

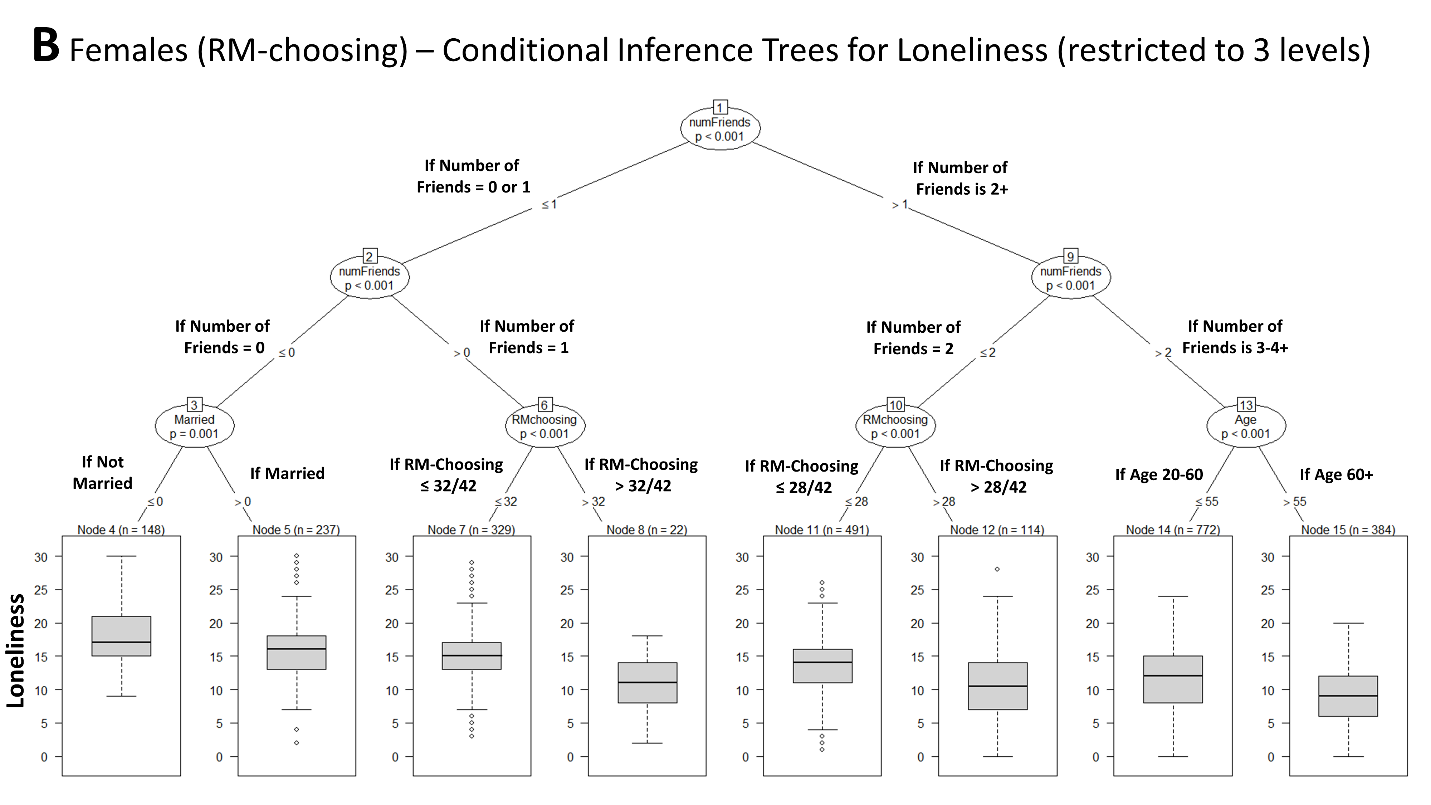


**Supplementary** **Figure S4: Conditional inference tree of major factors that predict loneliness in female sub-populations in Japan**

For female subjects only: the top three levels in importance of the best conditional inference tree for explanatory variables that maximize the ratio of the between-group variance to within-group variance for loneliness distributions within sub-populations in our general population sample. The highest branch has the most important variable for splitting, and then lower branches have descending importance. All explanatory variables that are included in Supplementary Figure S2 were input into the tree algorithm, other than in (A) choosing relational mobility was excluded as this tree just tested meeting relational mobility, and other than in (B) meeting relational mobility was excluded as this tree just tested choosing relational mobility.

*Sample Size Sensitivity & Robustness Checks*Relational mobility is a recently developed psychological construct, and our study is the first to look at within-culture association between individual perceptions of relational mobility and subjective loneliness. Thus, there is a lack of prior literature available to inform power analysis calculations of optimal survey sample sizes in this research topic. However, to our knowledge, our study is the largest relational mobility study for a general population sample in Japan by almost tenfold (the prior largest study was *N=*786 of Japanese Facebook users^4^ and did not examine within-culture loneliness). We use this advantage to perform several exploratory post-hoc analyses to test the sensitivity of our effect size and statistical significance results to sample size, to inform future work (Fig. S5-S7). We did not have pilot data to perform a preliminary power analysis and sample size calculation.

In our primary regression analysis (Table 2), the global effect size of our model was Cohen’s $f^{2}=0.449$, where Cohen’s $f^{2}\geq0.35$ is usually interpreted as a strong effect^5^. Within this well-performing regression model, the standardized regression coefficients give approximate localized effect sizes, but cannot be absolutely interpreted due to the variables being embedded in a multiple regression model, and due to limitations of the discrete Likert scales in the main variables of interest. However, the standardized coefficients allow the relative effect size between coefficients to be compared, here by comparing to the reference coefficient of number of close friends, known to be the dominant correlate of lower loneliness in literature, and also the largest standardized coefficient within our regression table (Table 2). The effect size (standardized regression coefficient) ratio of relational mobility (meeting) to number of close friends is 0.361, and the effect size ratio of relational mobility (choosing) to the number of close friends is 0.327. In comparison to three other large established correlates of loneliness in Japan and globally, the effect size ratio of marriage to number of close friends is 0.590, of gender to number of close friends is 0.512, and of age to number of close friends is 0.356. Thus, given the comparable relative effect sizes between relational mobility and important known socioeconomic correlates of loneliness, we expect perceptions of relational mobility to be of practical importance to policy makers.

We then explored how sensitive these effect size results are to sample size. Since our model has 35 predictor variables, it is reasonable to start this test with a minimum sub-sample size of at least several times above the number of degrees of freedom (e.g. a common rule of thumb in psychology research is to have a minimum sample size of $50+8\times number of predictors$ ^6^). We reran the main regression for randomly chosen samples of size 150, 300, 500, 750, 1000, 1500, and 2000 (our actual sample size is around 5000) within our full data set (repeating each sample size fitting with 20 randomly sampled runs per sample size, and averaging results over the 20 runs), to determine how the Cohen’s $f^{2}$, the *p-*value of the main regression coefficients (number of close friends and relational mobility), and the 95% confidence interval bounds of the main coefficients changed with sample size (Figures S5-S7). In this exploratory post-hoc analysis, a sample size of approximately 1000 was needed to achieve a stable Cohen’s $f^{2}\geq0.35$ (using adjusted R^2^ to correct for bias in the small sample sizes in the sweep^7^), for the Table 2 regression model. A sample size of approximately 500 and 1000 was needed to achieve 0.05 significance for relational mobility (meeting) and relational mobility (choosing) respectively. Similarly, a sample size of approximately 500 and 1000 was needed to achieve confidence interval ranges stably below zero for the relational mobility coefficients. Thus, our *N=*4,977 sample size was of the right order of magnitude, but well surpassed the minimum sample size needed to confirm our main effects.


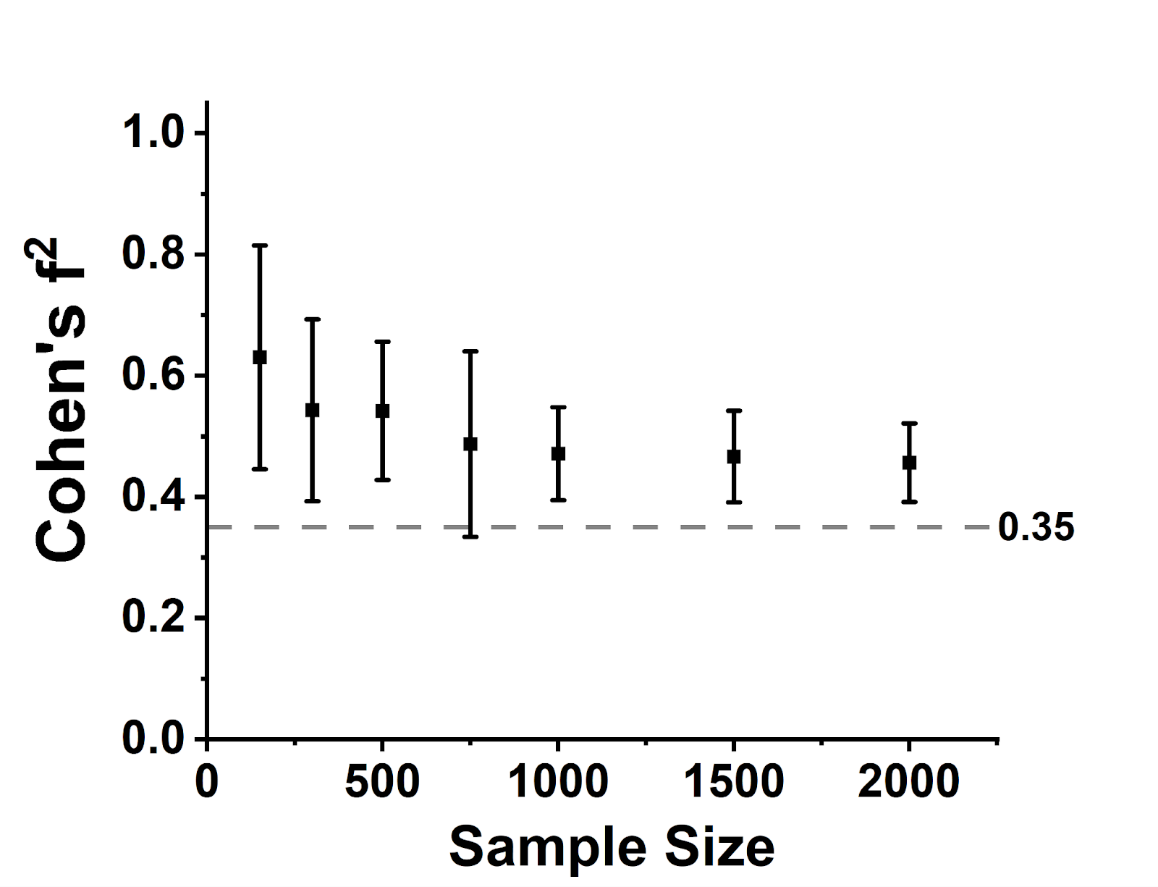


**Figure S5: Cohen’s *f^2^* (global effect size) for the Table 2 regression model versus sample size**
The average mean Cohen’s *f^2^* of the Table 2 regression model per sample size (*N*=20 randomly sampled runs per sample size). Plot error bars are 95% CI around the mean.


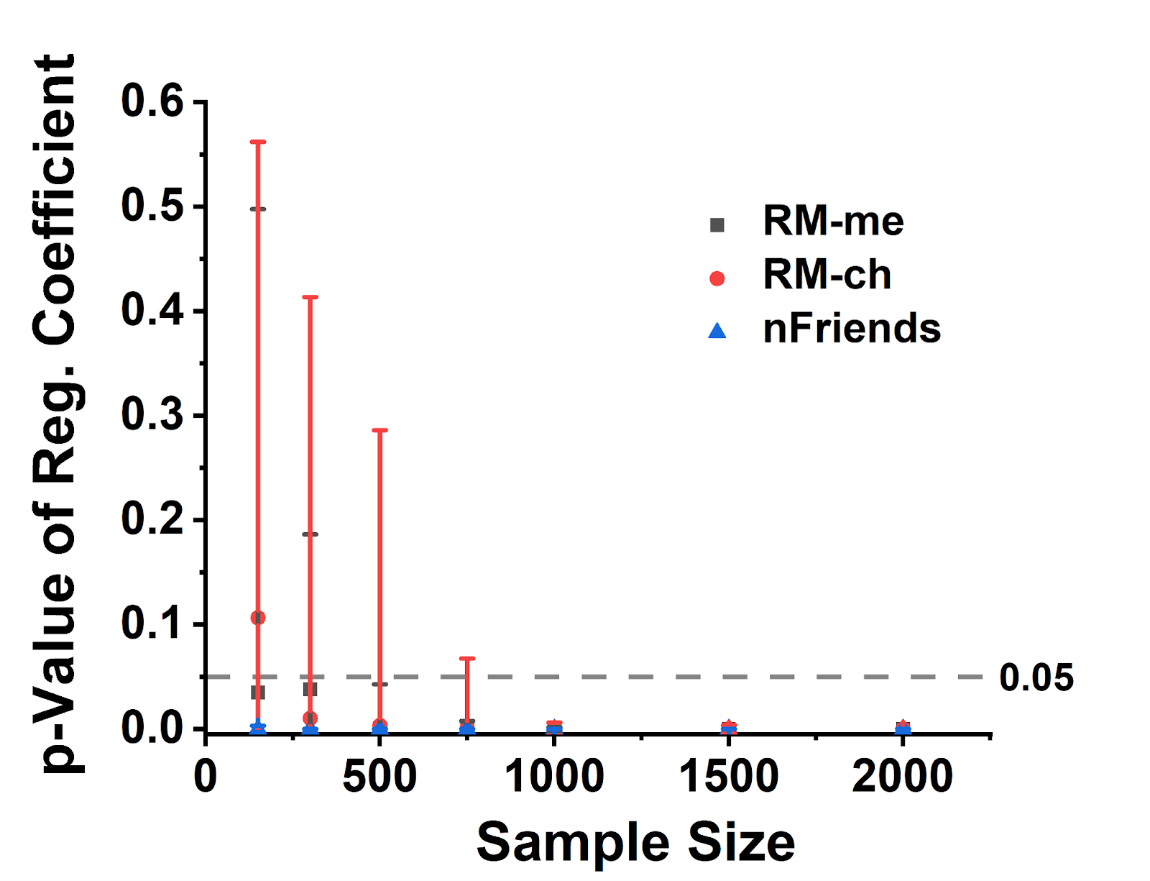


**Figure S6: p-Value of relational mobility and number of close friends within the Table 2 regression model versus sample size**

The average mean *p*-value of the standardized regression coefficient per sample size (*N*=20 randomly sampled runs per sample size) for relational mobility (meeting) (RM-me) and (choosing) (RM-ch), and number of close friends (nFriends). Plot error bars are 95% CI around the mean.


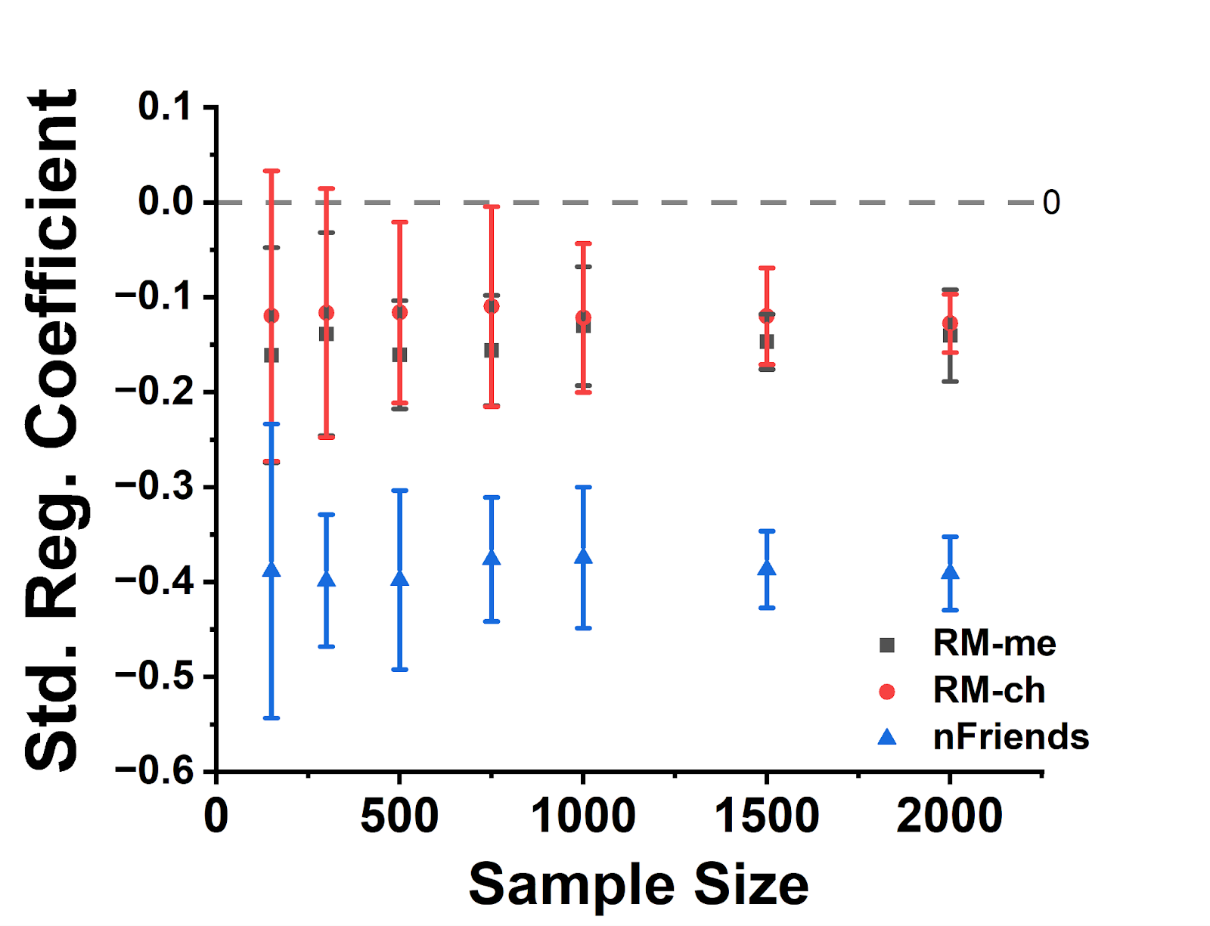


**Figure S7: Coefficient magnitude of relational mobility and number of close friends within the Table 2 regression model versus sample size**

The average mean standardized regression coefficient and upper/lower 95% confidence interval bounds per sample size (*N*=20 randomly sampled runs per sample size) for relational mobility (meeting) (RM-me) and (choosing) (RM-ch), and number of close friends (nFriends). Plot error bars are 95% CI around the mean, with the upper and lower confidence interval values being the mean upper and lower values across runs per sample size.

**References**

1. Heu, L. C., Hansen, N. & van Zomeren, M. Resolving the cultural loneliness paradox of choice: The role of cultural norms about individual choice regarding relationships in explaining loneliness in four European countries. *Journal of Social and Personal Relationships* **38**, 2053–2072 (2021).

2. Hothorn, T., Hornik, K. & Zeileis, A. Unbiased Recursive Partitioning: A Conditional Inference Framework. *Journal of Computational and Graphical Statistics* **15**, 651–674 (2006).

3. Johnstone, C. P., Lill, A. & Reina, R. Habitat loss, fragmentation and degradation effects on small mammals: analysis with conditional inference tree statistical modelling. (2014) doi:10.1016/J.BIOCON.2014.04.025.

4. Thomson, R. *et al.* Relational mobility predicts social behaviors in 39 countries and is tied to historical farming and threat. *PNAS* **115**, 7521–7526 (2018).

5. Cohen, J. *Statistical Power Analysis for the Behavioral Sciences*. (Routledge, 1988). doi:10.4324/9780203771587.

6. Morgan, B. L. & Voorhis, C. R. W. V. Understanding Power and Rules of Thumb for Determining Sample Sizes. *Tutorials in Quantitative Methods for Psychology* **3**, 43–50 (2007).

7. Keith, T. Z. *Multiple Regression and Beyond: An Introduction to Multiple Regression and Structural Equation Modeling*. (Routledge, 2019). doi:10.4324/9781315162348.
